# Platelets fine-tune effector responses of naïve CD4^+^ T cells via platelet factor 4-regulated transforming growth factor β signaling

**DOI:** 10.1007/s00018-022-04279-1

**Published:** 2022-04-18

**Authors:** Yanan Min, Long Hao, Xinguang Liu, Shuai Tan, Hui Song, Hao Ni, Zi Sheng, Natalie Jooss, Xuena Liu, Rickard E. Malmström, Yang Sun, Jianguo Liu, Hua Tang, Hao Zhang, Chunhong Ma, Jun Peng, Ming Hou, Nailin Li

**Affiliations:** 1 Department of Medicine-Solna, Cardiovascular Medicine Unit, J8:20, Karolinska Institute, Karolinska University Hospital-Solna, 171 76, Stockholm, Sweden; 2 Department of Hematology, Affiliated Hospital of Jining Medical University, Jining, China; 3 Department of Clinical Medicine, Shandong University of Traditional Chinese Medicine, Jinan, China; 4 Department of General Surgery, Affiliated Hospital of Jining Medical University, Jining, China; 5 Department of Hematology, Qilu Hospital of Shandong University, Jinan, China; 6 Department of Clinical Laboratory, Affiliated Hospital of Jining Medical University, Jining, China; 7 Department of Rheumatology, Qilu Hospital of Shandong University, Jinan, China; 8 Department of Medicine-Solna, Clinical Epidemiology Unit, Clinical Pharmacology Group, Karolinska Institute, Stockholm, Sweden; 9 Department of Laboratory Medicine, Clinical Pharmacology, Karolinska University Hospital-Solna, Stockholm, Sweden; 10 School of Basic Medicine, Department of Immunology and Shandong University-Karolinska Institutet Collaborative Laboratory, Shandong University Cheeloo Medical College, Jinan, China; 11 Shandong First Medical University and Shandong Academy of Medical Science, Institute of Immunology, Taian, China

**Keywords:** Platelets, Naïve CD4^+^ T cells, Effector cell responses, Platelet factor 4, Transforming growth factor β, Type II TGFβ receptor, Type III TGFβ receptor, Platelet–T cell interactions

## Abstract

**Background and aim:**

Platelets are an able regulator of CD4^+^ T cell immunity. Herein, the mechanisms underlying platelet-regulated effector responses of naïve CD4^+^ T (Tn) cells were investigated.

**Methods:**

Platelet–Tn cell co-cultures of human cells, genetically modified murine models, and high-throughput bioinformatic analyses were combined to elucidate molecular mechanisms of platelet-dependent regulation.

**Results:**

Platelets exerted sophisticated regulation on effector responses of type 1, 2, and 17 T helper (Th1/Th2/Th17) and regulatory T (Treg) cells, in time-, concentration-, and organ-dependent manners and with close cooperation of transforming growth factor β (TGFβ) and platelet factor 4 (PF4). PF4 at low concentrations reinforced TGFβ signaling by heteromerizing with type III TGFβ receptor (TGFBRIII), and subsequently enhanced TGFBRII expression and TGFβ signaling. High-concentration PF4 had, however, opposite effects by directly binding to TGFBRII, blocking TGFβ–TGFBRII ligation, and thus inhibiting TGFβ signaling. Furthermore, platelet depletion markedly hampered Treg and Th17 responses in the spleen but not in the lymph nodes, blockade of platelet–Tn cell contact diminished platelet effects, while spleen injection of PF4-immobilized microparticles in PF4-deficient mice mimicked platelet effects, suggesting the importance of direct platelet–Tn contact and platelet-bound PF4 for the optimal regulatory effects by platelets.

**Conclusion:**

Platelets exert context-dependent regulations on effector responses of Tn cells via PF4-TGFβ duet, suggesting new possibilities of platelet-targeted interventions of T cell immunity.

**Supplementary Information:**

The online version contains supplementary material available at 10.1007/s00018-022-04279-1.

## Introduction

Platelets are versatile cells. They not only play pivotal roles in hemostasis and thrombosis, but also actively engage in other physio-/pathophysiological processes, e.g., immunity, inflammation, and atherosclerosis [1–7]. It is known that platelets are closely involved in regulation of CD4^+^ T cell immune responses [4, 5, 8, 9]. They not only support T cell adhesion and recruitment [10, 11], but also exert distinct regulation on differentiation and/or activation of CD4^+^ T effector cells via multiple mediators [5, 9, 12]. Our earlier work demonstrated that platelets exert distinct dynamics of regulation on different CD4^+^ T effector cells, namely constant enhancements on Treg cell response but bi-phasic regulations on Th1 and Th17 activation [13].

Platelets store and release various bioactive mediators. Platelet factor 4 (PF4) is a chemokine stored in a large amount in platelet α-granules, and is released upon platelet activation [14]. It has been shown to influence both innate (e.g., macrophage polarization) and adaptive arms (e.g., Th cell proliferation and differentiation) of immunity [15, 16]. PF4 suppresses IL-2 production, and therefore inhibits proliferation of CD4^+^ T cells [17]. Moreover, PF4 stimulates Treg cell response, but attenuates Th17 differentiation and maintains Th17 homeostasis [18], in which PF4 exerts its Th17-inhibiting effects by impeding TGFβ signaling pathway, albeit the underlying mechanisms require further clarification.

Transforming growth factor β1 (TGFβ1) is another cytokine highly expressed in platelets, and has pleiotropic effects on various cell types, such as inhibiting Th1 but stimulating Treg/Th17 differentiation and proliferation [9, 17], through its widely expressed receptors, type I, II, III TGFβ receptors (TGFBRI, TGFBRII and TGFBRIII). Upon ligand binding, TGFBRII recruits and phosphorylates TGFBRI, which then phosphorylates downstream Smad2 and Smad3 (mother against decapentaplegic homolog 2 and 3). Phosphorylated Samd2/3 subsequently recruit Smad4, translocate to the nucleus, and regulate transcription of various genes [19]. TGFBRIII, also named betaglycan, is the most abundantly expressed TGFβ receptor. It has no kinase activities, but sequesters and presents TGFβ to TGFBRII. Moreover, the extracellular domain of TGFBRIII can be cleaved and released as soluble TGFBRIII (sTGFBRIII) that antagonizes TGFβ binding to TGFBRII [20].

Platelets are an important regulator maintaining functional balance of immunity and inflammation [4, 8, 9]. Platelet-derived TGFβ1 and PF4 act on the same subsets of CD4^+^ T cells with complex or even counteracting effects. There is evidence showing that PF4 interfered with TGFβ1 signaling [9, 18]. However, it remains undefined how these two platelet-derived mediators cooperate in their regulations on CD4^+^ T effector cell responses. Therefore, the present study investigated how platelets regulated effector cell responses of naïve CD4^+^ T (Tn) cells by TGFβ1 and PF4, using both in vitro and in vivo experimental models. We found that PF4–TGFβ duet exerted context-dependent regulation on effector responses of Tn cells. PF4 at low concentrations bound to TGFBRIII, facilitated TGFβ signaling, and thus enhanced effector responses of Tn cells. PF4 at high concentrations, however, competed TGFβ–TGFBRII ligation, and subsequently attenuated TGFβ signaling and effector cell responses. Furthermore, we revealed the importance of direct platelet–Tn contact and platelet surface-bound PF4 in the enhancement of Treg/Th17 responses and TGFβ1 signaling.

## Materials and methods

### Subjects

All healthy volunteers (aged 22–56 years, 25 males and 21 females) donated peripheral blood samples after given their written informed consent. All volunteers denied taking any medication during 2 weeks prior to blood sampling.

### Reagents

Reagents and antibodies used in the present study were summarized in Supplementary table I.

### Platelet and naïve CD4^+^ T cell isolation

For isolation and analysis of human platelets, peripheral blood samples were drawn using 10 ml vacutainer tubes containing 1 ml of 3.8% sodium citrate. Platelets were isolated immediately after blood collection. Whole blood samples were first centrifuged at 190 g (20 min). The upper two-third platelet-rich plasma (PRP) was taken and supplemented with prostaglandin I_2_ (PGI_2_, final concentration, 0.1 µg/ml), and re-spun at 190 g for 10 min. The supernatant was collected and re-span at 1000 g for 10 min. The pellet was re-suspended with Tyrode’s HEPES buffer containing 10 µM indomethacin and 0.1 µg/ml PGI_2_. Afterward, platelet pellet was re-suspended with basic culture medium and kept at room temperature for 30 min to allow the recovery of platelet responsiveness. All procedures were done at room temperature.

Peripheral blood mononuclear cells (PBMCs) were isolated using Ficoll-Paque® (Sigma). Tn cells were then isolated by negative selection using a magnetic separation kit according to manufacturer’s instructions. The purity of isolated Tn cells was > 95% as monitored by flow cytometry. Cells were then labeled with Tag-it violet according to manufacturer’s instructions.

### Cell cultures and genetic modifications of cell lines

Tn cells (10^6^/ml) were cultured in complete RPMI 1640 medium with or without washed platelets for up to 7 days. CD4^+^ T cells were activated by αCD3/αCD28 antibodies (5 µg/ml respectively).

HuT-78 cells were purchased from the American Type Culture Collection (ATCC, Manassas, VA, USA), cultured in IMDM medium containing 20% FBS, 100 U/ml penicillin, and 100 µg/ml streptomycin.

### Flow cytometry analyses

Flow cytometric Th1/Th2/Th17 phenotyping were performed by intracellular staining of corresponding IFNγ, IL-4, and IL-17, respectively, while Treg cell phenotyping combined CD25 surface expression and FoxP3 intracellular staining. For intracellular detection of IFNγ, IL-4, IL-17A, FoxP3, and phosphorylated Smad2/Smad3 expression, mouse or human T cells were activated with phorbol myristate acetate (50 ng/ml) and ionomycin (1 μg/ml; both form Sigma-Aldrich) in the presence of GolgiStop (1 μg/ml; BD Biosciences) for 5 h. Cells were immediately stained with Zombie Aqua™ dye, and then incubated with surface markers, fixed/permeabilized and stained for intracellular antigens with proper fluorochrome-conjugated antibodies. The data were acquired on BD FACSCanto™ II flow cytometer (BD Biosciences) or Galios® flow cytometers (Beckman-Coulter Corp.), and were analyzed with FlowJo software (TreeStar Inc.; Ashland, OR, USA).

### Detection of cytokines

Multiple cytokines (IL-2, IL-10, IL-4, IL-6, IFN-γ, TNF, and IL-17A) in supernatants or murine plasma were simultaneously measured using BD cytometric bead array (CBA) using a human or mouse Th1/Th2/Th17 kit. Acquisition was performed with a FACS Canto II flow cytometer. Quantitative results were generated using FCAP Array Software (BD Biosciences).

Concentrations of active TGFβ1, total TGFβ1, and sTGFBRIII in supernatants, as well as PF4 levels in murine plasma were detected by ELISA with corresponding ELISA kits. The murine plasma was 100-fold diluted before assay. To measure total TGFβ1, including both active and inactive latent TGF-β1 forms, samples were first acid-activated with sample activation kit 1 (with 1 N HCL) before assay.

### Protein–protein interaction (PPI) screening by high-throughput bimolecular fluorescence complementation technology (HT-BiFC) assay

The HT-BiFC screening was conducted with the support by MAGIGEN Biotechnology Co. (Guangdong, China). Briefly, a stable bait HTC-75 cells expressing PF4–YFPn were generated by infection with retrovirus for 48 h and selected with 300 µg/ml G418 for 10 days. Meanwhile, a pool of prey vectors were constructed from the human ORFeome v7.1 library, containing 18,414 human open reading frames (ORFs), using the Gateway recombination system and were tethered to the C-terminal fragment of YFP (YFPc). YPFc-tagged prey retroviruses were used to infect the stable YFPn-tagged PF4 bait cells for 48 h. Stable cell line co-expressing YFPn-tagged PF4 and YPFc-tagged prey was obtained by selection with 1 µg/mL puromycin for 3–5 days. HTC-75 cells infected with only YFPc-EV retroviruses were generated as the negative control. All procedures were performed with the Biomek 3000 Laboratory Automation Workstation (Beckman Coulter, Brea, CA, USA). The positive fluorescent cells were harvested and subjected to a next round of sorting until the desired positive rate (> 90%) was reached (supplementary Fig. 1). mRNAs were extracted and reverse-transcribed into cDNA, and were then identified through Illumina/Solexa sequencing [21].

### Immunofluorescent imaging

To create high intracellular PF4 and TGFBRIII concentrations for Bi-FC assay, a stable cell line expressing both truncated PF4–YFPn with 1–31 aa signal peptide deleted and truncated TGFBRIII (21-787)-YFPc or the truncated TGFBRII (23-166)-YFPc were generated as described above. The transfected 293 T cells were maintained in Dulbecco's Modified Eagle Media (DMEM) medium supplemented with 10% FBS at 37℃ in 5% CO_2_ (v/v). Forty-eight hours before imaging, 293 T cells were transfected with 1 µg plasmids encoding SFB- or GFP-tagged proteins for 6 h. For immunofluorescent staining, cells were fixed by 4% paraformaldehyde for 10 min at room temperature. Following blockade with 1% BSA for 1 h at room temperature, cells were then incubated with the rabbit anti-FLAG primary antibody for 1 h at room temperature. After that, SFB-tagged proteins were visualized by rhodamine-conjugated donkey anti-rabbit secondary antibody for 1 h, and cell nuclei were stained with 1 ng/ml DAPI for 2 min. Coverslips were mounted on slides with Fluoromount-G mounting medium, and examined and photographed with a 100 × oil immersion lens (Nikon Eclipse Ti-E, Japan).

### Immobilization of recombinant murine PF4 protein on amino microspheres

Glutaraldehyde activation of aminated microsphere beads and covalent immobilization of recombinant mouse PF4 (imPF4) was conducted according to manufacturer’s instructions. Briefly, aminated microspheres, 7.3 × 10^11^/ml, were activated using 2 ml of glutaraldehyde (EM grad, 8%, pH 7.4) overnight at room temperature with gentle end-to-end mixing. After completion of the activation process, the microspheres were washed with PBS for three times to remove unreacted glutaraldehyde. The imine double bounds were reduced by addition of the sodium borohydride (NaBH4, 1% in PBS). The reduction step was carried out at room temperature for 24 h under gentle stirring. Thereafter, the latex was cleaned by repetitive centrifugation/re-dispersion in PBS. The activated microspheres were then mixed with 25 μg of imPF4 protein or 20 μg BSA dissolved in 1 mL of PBS, pH 7.4. The mixture was gently and end-to-end rocked for 20 h at room temperature. Physically adsorbed imPF4 was removed from the surface by mixing microspheres with 0.1% (w/v) Tween 20 in PBS. The mixture was incubated at room temperature for 24 h with a slow shaking. Lastly, microspheres were spun down and re-suspended with 1 ml of PBS containing 10 mg/ml BSA, 0.1% NaN3 and 5% glycerol (pH 7.4) for storage at 4 °C.

After immobilization, the supernatant containing imPF4 was assayed for protein concentration. The amount of protein added before immobilization minus the amount in the supernatant represents the amount of protein bound to the beads. The estimated concentration of imPF4 on the surface of microspheres was 17.8 μg/ml.

For mice receiving PF4-immobilized microparticles (imPF4-MPs), 150 µl PBS containing 0.36 µg imPF4 was administered through spleen injection 1 day after T cell transfer.

### Mouse models

*Mice* Wide-type C57BL/6 mice were obtained from Beijing HFK bioscience (Beijing, China). All genetically modified mice were created on a C57BL/6 background. TCR transgenic OT-II mice that recognize OVA_323–339_ were purchased from the Jackson Laboratory (Shanghai, China) and backcrossed onto the mice with CD45.1 background, and served as donor mice in adoptive transfer of OT-II T cells. Platelet-specific TGFβ1-deficient (Plt-TGFβ^−/−^) mice were established by crossing mice carrying a “floxed” TGFβ1 allele (Tgfb1^flox^; Jackson Laboratory) to mice expressing Cre recombinase under control of the megakaryocyte-specific PF4 promoter (PF4CreTg^+^) (Model Animal Research Center of Nanjing University, Nanjing, China). PF4^−/−^ mice were generated using the CRISPR/Cas 9 system (Cyagen Biosciences Inc., Guangzhou, China). All mice were bred and housed in specific pathogen-free conditions at Shandong University. Eight- to twelve-week-old mice (male) were used as recipients for in vivo experiments.

*Murine model of platelet depletion and OT-II T cell adoptive transfer* Murine platelet depletion was carried out as previously describe [22]. Briefly, the recipient mice received either rat anti-mouse GPIbα/CD42b antibody R300 or non-specific rat IgG C301 (4 µg/g i.p. on day -2, followed by 2 µg/g i.p. on day 0, 3 and 5). Platelet counts were monitored on day 0, 1, 3, 5, and 7, and with platelet count of R300-treated (platelet-depleted) mice remained at < 10% of the control mice. To elucidate the impact of platelets on Tn cell responses in vivo, CD45.1 OT-II T cells were isolated from the spleens and LNs of OT-II mice using a CD4^+^ T cell isolation kit. OT-II T cells (4 × 10^6^/0.2 ml) were injected via the tail vein on day -1. Recipient mice are immunized *i.p.* with 130 µg of OVA in 200 ul of alum adjuvant on day 0. The mice were sacrificed at indicated checkpoints. Blood samples were collected by cardiac puncture for plasma cytokine analyses using a CBA assay. The spleens and the LNs were collected for mononuclear cell isolation and flow cytometric phenotyping assays as described previously [23]. Briefly, mononuclear cells were isolated by homogenization of whole spleens or LNs using 40 μm cell strainer (BD Biosciences). Cell suspensions were then overlaid on 5 ml of Ficoll-Paque (Weike Biotech Co; Shanghai, China) and centrifuged at 650 g for 30 min.

### Bioinformatic analyses

Protein sequence and functional information were obtained from the Uniprot Database (http://www.uniprot.org/). To further define the biological functions of PF4 interactome, PF4-interacting proteins were analyzed using DAVID Bioinformation Resources 6.8 [24] for gene ontology (GO) annotation, enrichment analysis and Kyoto Encyclopedia of Genes and Genomes (KEGG) pathway enrichment analysis. Additionally, a combination of the search tool for the retrieval of interacting gene/proteins (STRING) version 11.0 database [21] and Cytoscape version 3.8.0 [25] was used to explore and build protein–protein interaction (PPI) network.

### Statistics

Data presented are mean ± SEM. Student’s *t* test and one-way/two-way/RM ANOVA were used to analyze the difference between two or among multiple groups using GraphPad Prism 8.0 (San Diego, CA, USA), followed by corresponding multiple comparisons tests. *P* < 0.05 was considered statistically significant.

## Results

### Platelets influence effector cell responses of Tn cells in both cell concentration- and time-dependent manners

**Fig. 1:**
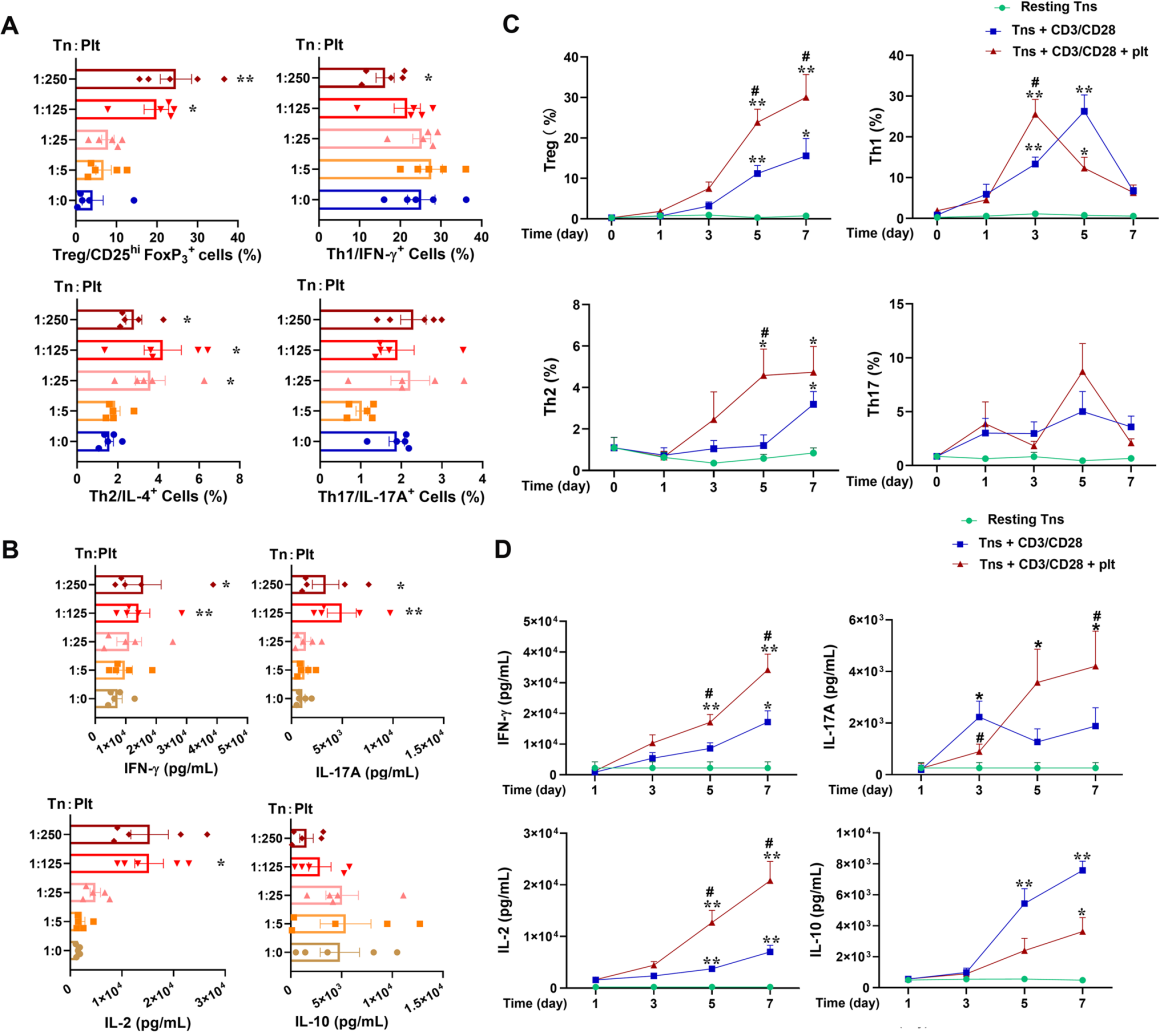
Platelets regulate effector cell responses of CD4^+^ naïve T cells in time- and concentration-dependent manners. Naïve CD4^+^ T (Tn) cells were cultured without or with αCD3/αCD28 stimulation and in the absence or presence of platelets (plts). **A**, **B** αCD3/αCD28-stimulated Tn cells were co-cultured with platelets at the Tn:plt ratios ranging from 1:0 to 1:250 for 5 days. Flow cytometric phenotyping of Treg/CD25^hi^FoxP3^+^, Th1/IFN-γ^+^, Th2/IL-4^+^, and Th17/IL-17A^+^ cells (**A**) and cytometric bead array (CBA) measurements of T effector cell cytokine levels in supernatants (**B**) were plotted. Mean ± SEM, *n* = 5; Differences of co-culture conditions were assessed using one-way ANOVA followed by Dunnett’s multiple comparison, *P < 0.05, **P < 0.01, as compared to Tn cells cultured alone. **C**, **D** Tn cells were cultured without () or with αCD3/αCD28-polyclonal stimulation () and in the absence () or presence () of platelets at the Tn:plt ratio of 1:250 during 7 days. CD4^+^ T cell phenotypings of Treg, Th1, Th2, and Th17 cells were assessed by flow cytometry (**C**). CD4^+^ T cell cytokine levels in the supernatants were measured by CBA (**D**). Mean ± SEM, *n* = 5. Comparisons were made by two-way ANOVA followed by Tukey’s multiple comparisons test. *P < 0.05, **P < 0.01, as compared to unstimulated Tn cells (); ***#P*** < 0.05, as compared to αCD3/αCD28-stimulated Tn cells cultured alone ()

Platelet–Tn cell co-cultures generally showed a platelet concentration-dependent enhancement or inhibition on effector cell responses of Tn cells during 5-day co-cultures (Fig. 1A). Thus, Treg/CD25^hi^FoxP3^+^ phenotype was elevated from Tn:plt ratio of 1:125 onward, while Th1/INFγ^+^ cells were suppressed at Tn:plt ratio of 1:250. These Tn:plt ratios are within the ranges in circulation and at the sites of inflammation/thrombosis, such as at the site of percutaneous coronary intervention that triggers massive platelet adhesion and subsequent leukocyte recruitment. The cytokines in the supernatant, which reflected the accumulated cytokine secretion during 5-day co-cultures, showed that platelets promoted the secretion of pro-inflammatory cytokines, e.g., IFNγ, IL-17A and IL-2 (Fig. 1B), as well as IL-6 and TNF (Supplementary Fig. 1A), largely in a platelet concentration-dependent manner, but that platelets tended to suppress IL-10 levels (Fig. 1B). Taken both CD4^+^ T cell phenotyping and cytokine production into consideration, Tn:Plt ratio 1:250 was thus chosen. To clarify the enhancements by platelets, the Th1 cytokine IFNγ in the supernatant of cell cultures was measured in a set of separate experiments. IFNγ of platelets cultured alone (56 ± 16 pg/ml) was neglectable as compared to the marked enhancement by platelets (IFNγ levels of αCD3/αCD28-stimulated CD4^+^ T cells from 5310 ± 2312 without to 13,973 ± 3847 pg/ml with platelet co-cultures; *n* = 8), indicating the effect as platelet-dependent enhancement of T cell activation rather than platelet-own production. Notably, cultured platelets underwent significant platelet activation and continuous secretion of CD4^+^ T cell-active mediators. Thus, TGFβ and PF4 levels were elevated proportionally to platelet concentrations, and Tn:Plt ratio 1:250 brought about high levels of TGFβ and PF4 (Supplementary Fig. 1C and 1D, upper panels), and the elevations were maintained during 7-day co-cultures (Supplementary Fig. 1C and 1D, lower panels). The persistently elevated TGFβ and PF4, together with direct platelet–Tn cell contact, jointly constituted a sustainable platelet regulation on Tn cell responses throughout the co-cultures, as evidenced by persistent elevations of the CD4^+^ T cell activation marker CD25 (Supplementary Fig. 1E).

The inconsistence between CD4^+^ T effector cell phenotyping and cytokine production (Fig. 1A, B), e.g., Th1 phenotypes and IFNγ levels, suggested that there might be a multi-phase regulation by platelets on Tn responses. Therefore, we next investigated how platelets dynamically regulate effector responses during 7-day co-cultures. As shown in Fig. 1C, platelets exerted time-dependent and distinct regulations on effector responses of Tn cells. Notably, platelet co-culture continuously promoted Treg and Th2 cell activation, while platelets enhanced Th1 activation on day 3 but suppressed on day 5.

When supernatant cytokine levels were monitored, αCD3/αCD28 stimulation markedly increased cytokine production, and platelets further elevated the levels of IFNγ, IL-17A, and IL-2 (Fig. 1D), as well as IL-6 and TNF (Supplementary Fig. 1B). Notably, the platelet-elevated cytokine levels in the supernatant reflected the accumulation of cytokine secretion by CD4^+^ T effector cells during 5-day co-cultures. Contrasting to continuously elevated Treg phenotypes, platelet co-cultures were associated with lower IL-10 levels (Fig. 1D). This is probably because PF4 can inhibit IL-10 production of CD4^+^CD25^−^ non-regulatory T cells that produce much higher amount of IL-10 than CD4^+^CD25^++^ regulatory T cells [17]. Albeit platelets and PF4 have been shown to increase Treg cell IL-10 production [12, 17], the increments were much less than the decrements seen among CD4^+^CD25^−^ non-regulatory T cells [17].

Taken together, platelet-regulated effector cell responses of Tn cells depended on platelet concentrations, effector cell types, as well as co-culture time-course. Platelets persistently boosted Treg/Th2 responses, while casted bi-phasic regulation on Th1/Th17 responses.

### Platelets enhance CD4^+^ T cell responses in vivo via both soluble factors and cell-to-cell contact

**Fig. 2:**
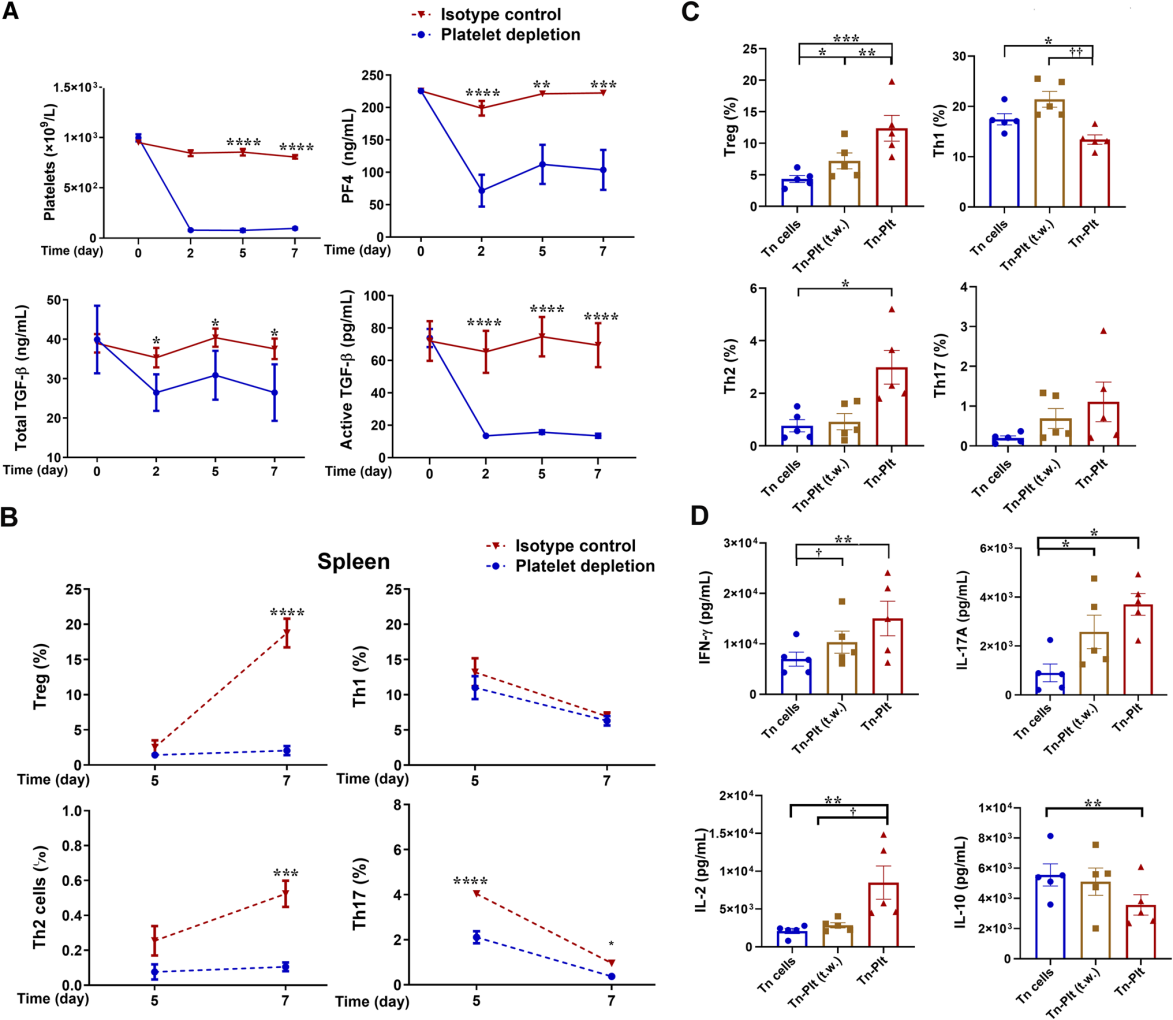
Platelets influence CD4^+^ Tn cell responses via both soluble mediators and cell-to-cell contact. **A**, **B** OTII T cell adoptive transfer and platelet depletion were employed to elucidate platelet-regulated CD4^+^ T effector cell responses. CD4^+^ T cells were isolated from lymphoid organs of CD45.1 OT-II mice and transferred into CD45.2 mice on day -1, followed by intraperitoneal injection of the rat anti-mouse GPIbα/CD42b antibody R300 (4 µg/g i.p. on day -2, followed by 2 µg/g i.p. on day 0, 3 and 5; blue circles and lines,) or the non-specific rat IgG antibody C301 (red triangles and lines,). Platelet-depleted and control mice were sacrificed on day 2, day 5 and day 7. Blood samples were collected on indicated days. Circulating platelets were counted. Plasma levels of PF4 (upper right plot), the total (lower left plot) and active TGFβ1 (lower right plot) were detected by ELISA (**A**). CD4^+^ T helper cell phenotyping of mononuclear cells from the spleen were gated on CD45.1^+^CD4^+^ T cells after eliminating cell debris and dead cells. CD4^+^ T cell phenotyping was performed for Treg/FoxP3^+^, Th1/IFN-γ^+^, Th2/IL-4^+^, and Th17/IL-17A^+^ (**B**). **C**, **D** CD4^+^ Tn cells were stimulated with αCD3/αCD28 antibodies, and cultured in the absence (blue circles and bars) or presence of platelets (Tn:plt = 1:250) without (red triangles and bars) or with transwell inserts (brown squares and bars; transwell membrane aperture 0.4 μm) for 5 days. CD4^+^ T effector cell phenotyping was assayed by flow cytometry (**C**), and cytokine levels in the supernatants were assessed by a CBA assay (**D**). Data were expressed as mean ± SEM; *n* = 5. Statistical analyses, for the data presented in panels A and B, were performed using two-way ANOVA followed by Sidak’s multiple comparison test. *P < 0.05, **P < 0.01, ***P < 0.001, ****P < 0.0001. For data analyses in **C**, **D**, RM one-way ANOVA followed by Holm–Sidak’s multiple comparisons test were applied. *P < 0.05, **P < 0.01, ***P < 0.001. Paired t tests were also conducted between groups, †*P* < 0.05, ††*P* < 0.01

The above results highlighted that platelets exert complex influences on Th1/Th2/Th17 and Treg responses of Tn cells in vitro. We next asked if and how platelets regulated CD4^+^ T effector cell responses in vivo using a murine model combining OT-II CD4^+^ T cell adoptive transfer and platelet depletion (Supplementary Fig. 2A). The latter was achieved using the rat anti-mouse GPIbα/CD42b antibody R300 [22], which triggers a rapid platelet destruction by macrophages and thus marked reductions in platelet counts and plasma PF4 (Fig. 2A). Platelet depletion reduced plasma PF4 levels less dramatically, which reflected a gradual clearance of PF4 in plasma and residual production of PF4 from non-platelet cells, such as monocytes/macrophages [26]. It should be noted that the active form of TGFβ1, which exerts TGFβ1 activities and is detected by an active form-specific antibody, in systemic circulation dramatically decreased along with platelet depletion. The total TGFβ1 was not proportionally decreased (Fig. 2A). The data indicated that platelets play a crucial role in activation of latent TGFβ1.

The transgenic OT-II CD4^+^ T cells express ovalbumin (OVA)-specific TCRs, and can be specifically activated upon OVA immunization [27]. Hence, OVA challenge evoked marked effector cell activation/differentiation on Day 5 and 7 among spleen-resided OT-II CD4^+^ T cells (Fig. 2B) of control mice. Platelet depletion largely abolished Treg and Th2 responses, while it also impeded Th17 response in the spleen. However, platelet depletion had no influence on OVA-stimulated Th1/Th2/Th17 responses of lymph node (LN)-resided OT-II CD4^+^ T cells (Supplementary Fig. 2B). As expected, due to the small mass of transferred OT-II T cells, plasma levels of the related cytokines were not altered (Supplementary Fig. 2C).

The distinct influences of platelet depletion on CD4^+^ T effector cell responses in two lymphoid organs indicate that platelet–T cell contact, which exists in the spleen but is largely absent in the LNs, may be important in platelet-regulated effector cell responses. Hence, platelet–Tn cell co-cultures were performed without or with transwells for 5 days. Figure 2C shows that direct platelet–Tn co-cultures enhanced Treg cell activation but attenuated Th1 responses upon αCD3/αCD28 stimulation, and that the regulations were significantly reduced in transwell co-cultures, indicating that the regulations were driven by both platelet-released soluble mediators and direct platelet–T cell contact. Analyses of effector cell cytokines further supported the importance of platelet–Tn cell contact. Thus, elevated IL-2 and decreased IL-10 levels in direct platelet co-cultures were dismissed by the chamber septum (Fig. 2D), and the elevations of proinflammatory IL-6 and TNF were also decreased (Supplementary Fig. 2B).

### Impacts of platelet-derived TGFβ on effector cell responses

**Fig. 3:**
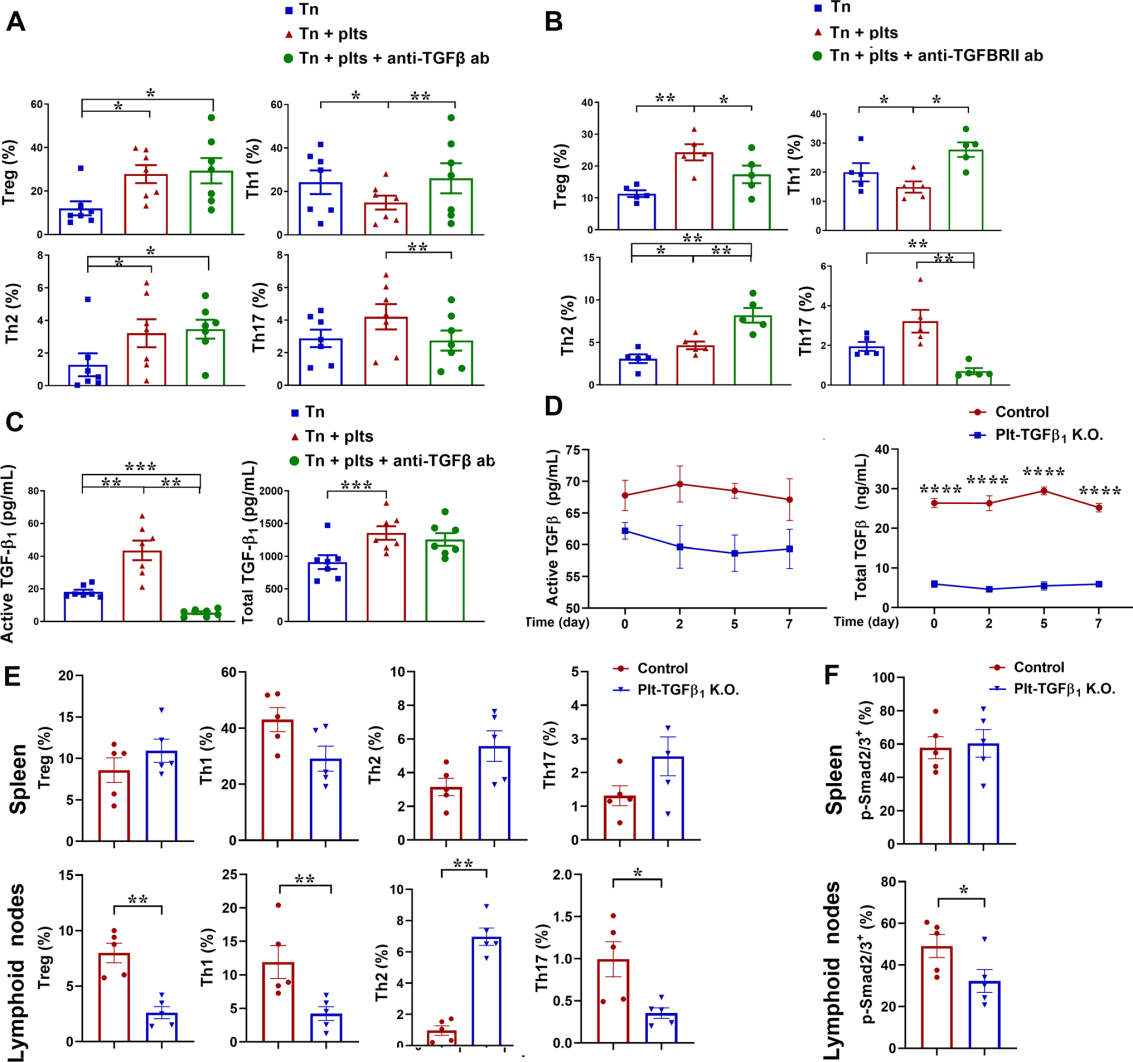
Impacts of platelet-derived TGFβ1 on naïve CD4^+^ T cell responses. **A**, **C** Naïve CD4^+^ T cells were stimulated with αCD3/αCD28 antibodies in the absence (blue squares) or presence (red triangles and green circles) of platelets (Tn:plt = 1:250), and cultured for 5 days with () or without () a TGF-β neutralizing antibody (20 µg/ml). Data plotted are flow cytometric phenotyping of T helper cells (**A**) and the total/active TGFβ1 levels in the supernatants as measured by ELISA (**C**), *n* = 7. Panels B: Naïve CD4^+^ T cells were stimulated with αCD3/αCD28 antibodies in the absence () or presence () of platelets (Tn:plt = 1:250), and cultured with () or without () a TGFBRII-blocking antibody (15 µg/ml) for 5 days. Flow cytometric phenotyping of T helper cells were plotted (*n* = 5). **D**–**F** Platelet-specific TGFβ1 knockout mice or control mice were adoptively transferred with CD4^+^ T cells from CD45.1 OT-II mice, followed by OVA challenge. Plasma levels of the total and active TGFβ were monitored by ELISA during 7 days (**D**; *n* = 5). Mononuclear cells were isolated from the spleen of recipient mice and assayed for flow cytometric phenotyping of Treg, Th1, Th2, and Th17 cells (**E**; *n* = 5), as well as phosphorylation levels of Smad2/3 of OT-II T cells (**F**; *n* = 5). Data were presented as mean ± SEM. For data presented in **A**–**C**, the comparisons among the treatments were performed using RM ANOVA followed by Tukey’s multiple comparison test. For data in **D**, comparisons between the groups were performed using two-way ANOVA followed by Sidak’s multiple comparison test. For data presented in **E**–**F**, Mann–Whitney test was used. *P < 0.05, **P < 0.01, ***P < 0.001, ****P < 0.0001

As TGFβ1 is a key soluble mediator of platelet-regulated CD4^+^ T effector responses [9, 13], its action deserved delineation in platelet–Tn cell co-cultures. The impacts of platelet-derived TGFβ on Tn cell responses were first investigated using a TGFβ-neutralizing antibody. Platelet-enhanced Treg and Th2 cell activation of αCD3/αCD28-stimulated Tn cells were not inhibited by TGFβ neutralization (Fig. 3A). In contrast, platelet-inhibited Th1 and platelet-enhanced Th17 responses of Tn cells were abolished by TGFβ neutralization (Fig. 3A), which also decreased IL-17A production (supplementary Fig. 3). The control rabbit IgG of TGFβ-neutralizing antibody did not influence CD4^+^ T effector cell responses (e.g., Th1 cells were 5.3 ± 0.7% without and 5.9 ± 1.2% with control IgG; *n* = 4). Notably, platelet co-culture markedly elevated both the total and, especially, active TGFβ1 levels; TGFβ neutralization almost eliminated active TGFβ1, confirming its efficiency and specificity for TGFβ1 activity silencing, but kept the total TGFβ1 levels largely unchanged (Fig. 3C).

To further elucidate the influence of TGFβ1 signaling on platelet-regulated CD4^+^ T cell responses, a type 2 TGFβ receptor (TGFBRII)-blocking antibody was used to block TGFβ1 signaling. Compared to TGFβ neutralization, TGFBRII blockade demonstrated more profound effects. Thus, TGFBRII blockade (Fig. 3B) counteracted platelet regulation on Treg and Th1 responses, and potentiated Th2 responses. TGFBRII blockade not only completely wiped off the platelet-enhanced Th17 response but also suppressed αCD3/αCD28-stimulated Th17 activation. In accordance with the changes in effector cell phenotypes, TGFBRII blockade more markedly elevated pro-inflammatory IFNγ, IL-2 and TNF levels, but suppressed IL-10 secretion (supplementary Fig. 4).

To confirm the impacts of platelet-derived TGFβ on Tn cell responses in vivo, OT-II CD4^+^ T cell adoptive transfer was performed in platelet-specific TGFβ1 knockout (plt-TGFβ^−/−^) mice, in which both levels of the total TGFβ1 and active TGFβ1 were markedly lower than those of control mice (Fig. 3D). For OT-II T cells from the spleen (Fig. 3E, upper panels), OVA-challenged Treg, Th1, Th2, and Th17 cell responses were, unexpectedly, not subverted in plt-TGFβ^−/−^ mice, as compared to the control mice. For OT-II CD4^+^ T cells from the LNs (Fig. 3E, lower panels), OVA-induced Treg, Th17, and Th1 cell responses were markedly reduced, while Th2 responses were much enhanced in plt-TGFβ^−/−^ mice. Figure 3F also shows that p-Smad2/3 signaling was lower in OT-II CD4^+^ T cells from the LNs (lower panel) but not from the spleen (upper panel) of plt-TGFβ^−/−^ mice.

Taken together, above observations give three key messages, (i) platelets are critical for TGFβ1 activation, seen as the dramatic reduction of active TGFβ1 in platelet-depleted mice (Fig. 2A); (ii) some other platelet-derived mediator(s) seem to engage in TGFβ-regulated Tn cell responses, as TGFBRII blockade produced more profound effects (Fig. 3B) than neutralization of TGFβ per se (Fig. 3A); (iii) platelet–Tn cell contact has important impacts on platelet-regulated Tn responses, because transwell co-cultures attenuated platelet effects (Fig. 2C, D).

### PF4 regulates effector cell responses by modulating TGFβ1 signaling

**Fig. 4:**
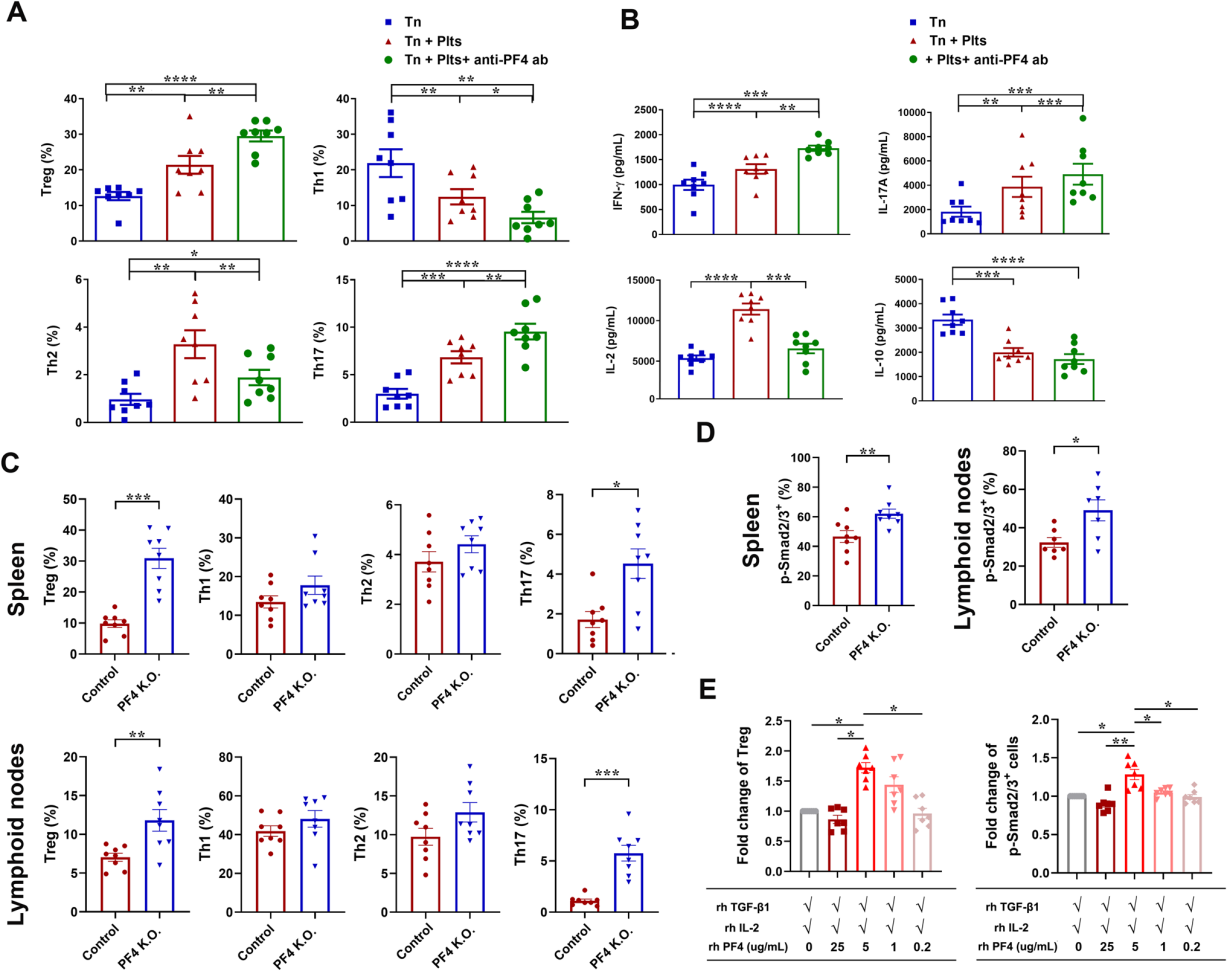
Impacts of PF4 on naïve CD4^+^ T cell responses. **A**, **B** Naïve CD4^+^ T cells were stimulated with αCD3/αCD28 antibodies in the absence (blue squares) or presence (red triangles and green circles) of plts (Tn:plt = 1:250), and cultured for 5 days with () or without () a PF4 neutralizing antibody (25 µg/ml). Flow cytometric phenotyping of Treg, Th1, Th2, and Th17 cells were presented in **A**. CD4^+^ T cell cytokine (**B**) in the supernatants were measured by CBA assay; *n* = 8. **C**, **D** PF4 knockout mice and their littermate (control) mice were adoptively transferred with CD4^+^ CD45.1 OT-II T cells, followed by OVA challenge. After 7-day incubation, mononuclear cells were isolated from the spleen and the lymph nodes of recipient mice, and assayed for flow cytometric phenotyping of Treg, Th1, Th2, and Th17 cells in CD45.1 OT-II T cells (**C**; *n* = 8), as well as phosphorylation levels of Smad2/3 of OT-II T cells (**D**; *n* = 8). **E** Naïve CD4^+^ T cells were stimulated with αCD3/αCD28 antibodies in the presence of rhTGFβ1 (5 ng/mL) and rhIL-2 (100 IU/mL) and without or with rhPF (0.2–25 µg/ml) for 5 days. Treg cell responses and TGFβ signaling/pSmad2/3 were monitored by flow cytometry. Data were presented as mean ± SEM. For data presented in **A**, **B**, the comparisons among the treatments were performed using RM ANOVA followed by Tukey’s multiple comparison test. For data in **C** and **D**, Mann–Whitney test was applied. For data presented in panel E, Friedman test was followed by Dunn’s multiple comparisons test, *n* = 6. *P < 0.05, **P < 0.01

PF4 is a known CD4^+^ T cell-active chemokine [9, 17, 28]. As stated above, other platelet-derived mediators are involved in and potentiate TGFβ signaling, we thus wondered if platelet PF4 contributes to platelet-regulated Tn responses. Figure 4A shows, surprisingly, that platelet-enhanced Treg- and Th17 responses of αCD3/αCD28-stimulated Tn cells were further enhanced by PF4 neutralization, and that platelet inhibition on Th1 responses was further strengthened. In contrast, PF4 neutralization attenuated platelet-boosted Th2 cell response (Fig. 4A), and exerted complex regulations on cytokine production. Thus, PF4 neutralization enhanced IFNγ and IL-17A production (Fig. 4B), but counteracted platelet-enhanced IL-6 and TNF production (supplementary Fig. 4A). The effects were not achieved by TGFβ activation, as PF4 neutralization affected neither active nor the total TGFβ levels (supplementary Fig. 4B). In contrast, supplementation of recombinant PF4 (rhPF4, 5 µg/ml) alone did not alter CD4^+^ T effector cell responses (supplementary Fig. 4C and 4D).

To further investigate the impact of PF4, a PF4 knockout murine strain was established, and OT-II CD4^+^ T cell adoptive transfer was performed. Similar to PF4 neutralization in vitro, OVA-evoked Treg and Th17 responses of CD4^+^ T cells from both the spleen and LNs were markedly enhanced in PF4^−/−^ mice (Fig. 4C). Furthermore, p-Smad2/3 levels of OT-II T cells were elevated, indicating that TGFβ signaling was boosted in PF4^−/−^ mice (Fig. 4D).

To investigate the discrepancy in the regulations of effector responses by PF4 neutralization/deficiency and by PF4 supplementation, gradient concentrations of rhPF4, mimicking plasma level (0.2 µg/ml), submaximal platelet activation (1 µg/ml), serum level (5 µg/ml), and a local level at thrombosis sites (25 µg/ml), were added to αCD3/αCD28-stimulated Tn cells cultured with rhTGFβ1 (5 ng/mL) and rhIL-2 (100 IU/mL). PF4 from 0.2–5 µg/ml showed a concentration-dependent enhancement on Treg cell responses. PF4 at 25 µg/ml, however, dismissed its enhancement. Moreover, changes of Treg cell responses were mirrored by TGFβ signaling/pSmad2/3 levels (Fig. 4E). Taken together, these results indicate that PF4 exerts complex regulation on CD4^+^ T effector cell responses of Tn cells, and that PF4 seems to exert its effects via regulating the action of TGFβ1 and/or the TGFβ signaling. This is because the most marked regulations by PF4 interventions, either by PF4 neutralization, supplementation, or knock-out, were seen in TGFβ-driven Treg and Th17 cell responses.

### Characterization of the PF4 interactome

The above observations indicate the importance of identifying the intervention site(s) of the TGFβ signaling by PF4. Therefore, high-throughput bimolecular fluorescence complementation (HT-BiFC) assay [29] was applied within a human ORFeome library containing more than 18,000 human cDNA clones [30]. The sifted-out proteins with their total reads were listed in supplementary table 1. KEGG pathway analyses showed that metabolic and endocytosis pathways were among the most enriched eleven pathways of the PF4-interacting proteins (Supplementary Fig. 5A/upper panel). GO analyses were also performed to characterize biological attributes for the PF4-interacting proteins. The top fifteen PF4-interacting protein groups within the “biological process” (Supplementary Fig. 5A/lower panel) revealed that PF4-interacting proteins were significantly enriched in multiple biological functions (*p* < 0.05), notably immune effector process (*p* = 1.68 × 10^–6^) and immune system process (*p* = 2.38 × 10^–5^).

Protein–protein interaction (PPI) network of PF4-interacting proteins was explored using the STRING database (https://string-db.org). By applying a medium confidence (*p* > 0.4), 105 (51%) of the PF4-interacting proteins formed 136 PPI edges and were tied to a single large network with a PPI enrichment (*p* < 0.05; Supplementary Fig. 5B), indicating that the interacting proteins were biologically connected as a network with a significantly greater number of interactions rather than as a random set of proteins.

### PF4 acts as an accelerator of TGFβ signaling pathway via binding to glycosaminoglycan-rich region of TGFBRIII

**Fig. 5:**
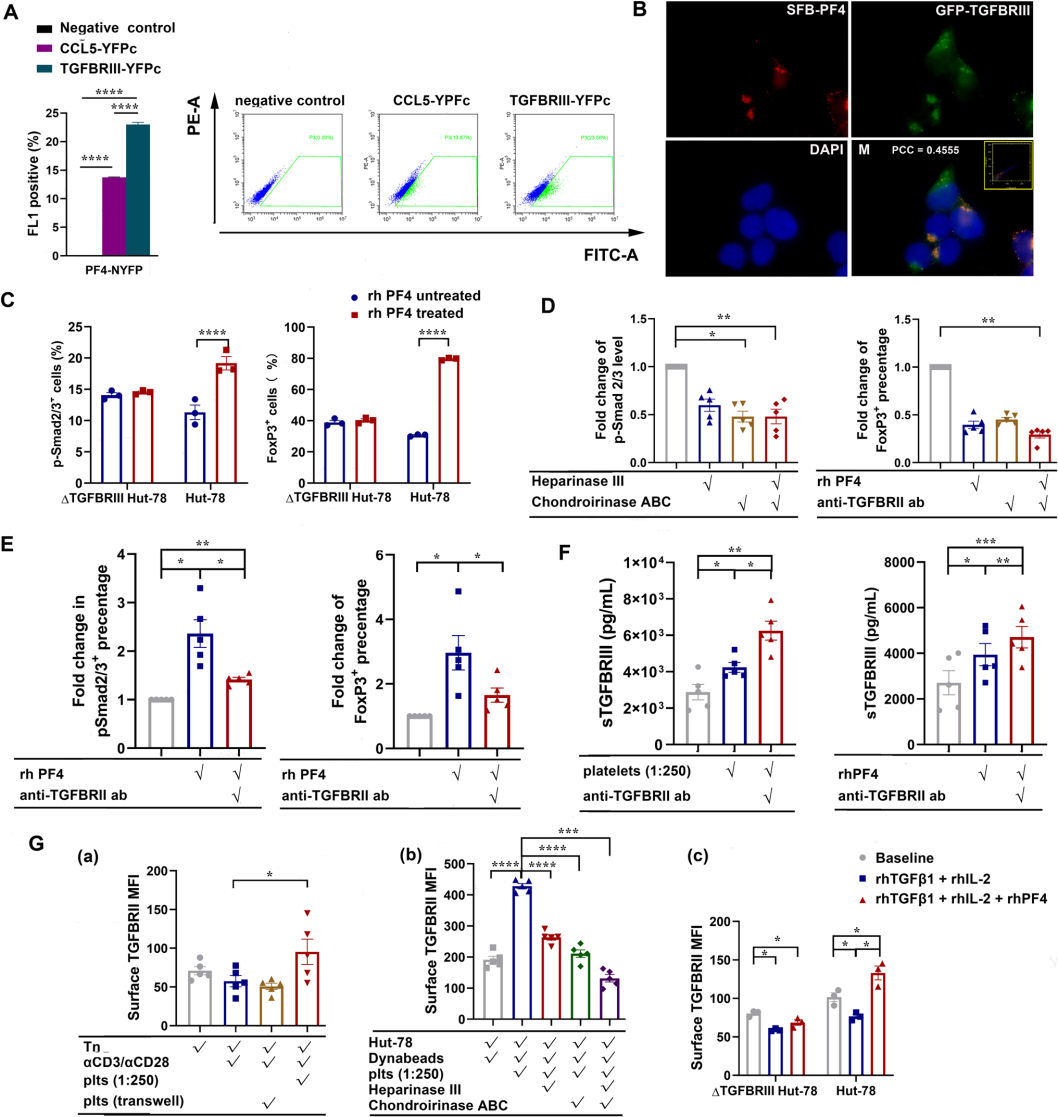
PF4 interactome and its interactions with and impacts on TGFβ signaling of CD4^+^ T cells. **A** BiFC confirmation of PF4-TGFBRIII heteromerization. HTC75 cells expressing both PF4–YFPn and YFPc-tagged TGFBRIII/CCL5/RANTES (positive control)/non-fused YFPc (negative control) were phenotyped for their yellow fluorescence signals; *n* = 3; one-way ANOVA followed by Holm–Sidak´s multiple comparison test was performed. **B** The localization of TGFBRIII and PF4 were revealed by immunostaining. 293 T cells were transfected with plasmids encoding GFP-tagged TGFBRIII and with plasmids encoding SFB-tagged PF4. Nucleus was visualized by DAPI staining. M, merged. Scatterplot of red and green pixel intensities was shown on the upper right corner of the merged picture. Pearson’s correlation coefficient (PCC) and scatterplot were calculated using ImageJ software from the compressed picture. **C** Wild type and TGFBRIII knockout (ΔTGFBRIII) HuT-78 cells were stimulated with αCD3/αCD28-coated Dynabeads (bead:cell ratio at 2:1) for 48 h, and then supplemented without or with rhPF4 (5 µg/mL) for a further 72-h culture. P-Smad2/3 and FoxP3 expression levels in HuT-78 cells were assessed by flow cytometry; *n* = 5; comparison between different treatment within the same cell line was performed two-way ANOVA followed by Sidak´s multiple comparison test. **D** HuT-78 cells were pretreated with vehicle, heparinase, and/or chondroitinase for 2 h, and then stimulated with αCD3/αCD28-coated Dynabeads for 48 h, and then further cultured in the absence or presence of rhPF4 (5 µg/mL) for 72 h. P-Smad2/3 and FoxP3 expression levels of HuT-78 cells were assessed by flow cytometry; *n* = 5; Friedman test were performed followed by Dunn´s multiple comparisons test. **E** Hut-78 cells were stimulated with αCD3/αCD28-coated Dynabeads for 48 h, and then cultured without or with rhPF4 (5 µg/mL) and in the absence or presence of TGFBRII-blocking antibody for 72 h; *n* = 5; the comparisons among the treatments were performed using RM ANOVA followed by Tukey’s multiple comparisons test. **F** Hut-78 cells were stimulated with αCD3/αCD28-coated Dynabeads for 48 h, and then in the absence or presence of platelets for 72 h (Hut-78:plt = 1:250) (left bar chart) and without or with rhPF4 (5 µg/mL). Soluble TGFBRIII levels in the culture media were analyzed by ELISA; *n* = 5; the comparisons among groups were performed using RM ANOVA followed by Tukey’s multiple comparison test. **G** (a) αCD3/αCD28-stimulated Tn cells were co-cultured with platelets (Tn:plt = 1:250) without or with a transwell for 5 days. Tn cell TGFBRII expression was assessed by flow cytometry, *n* = 5. (b) HuT-78 cells were pre-treated with vehicle, heparinase, or chondroitinase for 2 h, and then stimulated with αCD3/αCD28-coated Dynabeads for 48 h. The cells were further cultured in the absence or presence of platelets for 72 h. TGFBRII expression of HuT-78 cells was assessed by flow cytometry; *n* = 5. The comparisons among treatments were performed using RM ANOVA followed by Dunnett’s multiple comparisons test. (c) Wild type and TGFBRIII knockout (ΔTGFBRIII) HuT-78 cells were stimulated with αCD3/αCD28-coated Dynabeads (bead:cell ratio at 2:1) for 48 h, and cultured without or with the supplementation of rhTGFβ (5 ng/mL)/rhIL-2 (100 IU/mL) or rhTGFβ (5 ng/mL)/rhIL-2 (100 IU/mL)/rhPF4 (5 µg/mL). TGFBRII expression of the cells were assessed by flow cytometry; *n* = 3; comparisons among treatment groups were performed using Tukey´s multiple comparisons test. Data were presented as mean ± SEM. *P < 0.05, **P < 0.01, ***P < 0.001, ****P < 0.0001

Among the top 200 PF4-interacting proteins, TGFBRIII, a co-receptor for the TGFβ superfamily and abundantly expressed on T cells, emerged as the most likely partner of PF4 in correlation with its TGFβ signaling intervention activity. Therefore, BiFC assay was carried out to confirm HT-BiFC assay-suggested PF4-TGFBRIII interaction. CCL5/RANTES-CXCL4/PF4 hetero-dimerization [31] was chosen as the positive control. Figure 5A shows that the cells co-transfected with PF4–YFPn and TGFBRIII–YFPc vectors demonstrated a higher positive rate (23.0% ± 0.4%) than PF4–YFPn–CCL5–YFPc co-transfected cells (13.8% ± 0.0%), suggesting that TGFBRIII-PF4 heteromerization was stronger than CCL5-PF4 heteromerization (*p* < 0.0001). Indeed, immunofluorescence imaging (Fig. 5B) shows that PF4 and TGFBRIII fluorescence co-localized in TGFBRIII-C-GFP (green fluorescence protein) and PF4-N-SFB (S protein–FLAG–streptavidin-binding peptide) co-expressed 293 T cells, confirming PF4–TGFBRIII interaction.

To further investigate PF4–TGFBRIII cooperation in platelet-regulated Tn responses, a TGFBIII knockout (ΔTGFBRIII) HuT-78 cell line was generated. After 48-h stimulation with αCD3/αCD28-coated Dynabeads (bead:cell ratio at 2:1), rhTGFβ1 (5 ng/mL) and rhIL-2 (400 IU/mL) were added without or with rhPF4 (5 µg/mL) for a further 72-h culture. Ligation of TGFβ and its receptors trigger phosphorylation of the TGFβ signal transmitter Smads and subsequently the expression of the transcription factors, e.g., FoxP3. Hence, Fig. 5C demonstrates that rhPF4 supplementation markedly enhanced p-Smad2/3 levels and FoxP3 expression in HuT-78 cells, but had no effects in ΔTGFBRIII HuT-78 cells, indicating that TGFBRIII is crucial in PF4-enhanced TGFβ1 signaling and downstream FoxP3 expression.

The above findings prompted the next question of how TGFBRIII and PF4 interact. TGFBRIII is rich in heparan sulfate (HS) and chondroitin sulfate glycosaminoglycans (GAG) [32, 33], to which PF4 contains their binding domains [34]. We thus hypothesized that PF4 might bind to the GAG-rich regions of TGFBRIII and subsequently enhance TGFβ signaling. To confirm the hypothesis, HuT-78 cells were pretreated with vehicle, heparinase, and/or chondroitinase for 2 h, and then stimulated with αCD3/αCD28-coated Dynabeads in the presence of rhPF4, rhTGFβ and rhIL-2. Figure 5D shows that rhPF4 supplementation increased p-Smad2/3 and FoxP3 levels of HuT-78 cells, and that enzymatic GAG digestion substantially decreased PF4 effects.

TGFBRIII has no kinase activity itself, but can bind TGFβ and present it to TGFBRII [35, 36]. To prove that PF4–TGFBRIII interaction facilitates TGFβ signaling via TGFBRII, we showed that TGFBRII blockade markedly reduced rhPF4-enhanced Smad2/3 phosphorylation and FoxP3 expression (Fig. 5E). Notably, the extracellular domain of TGFBRIII can be cleaved and act as a TGFβ antagonist by preventing TGFβ ligation to TGFBRII [37, 38]. Thus, we asked whether PF4–TGFBRIII-binding influences sTGFBRIII release and thus inhibits TGFβ signaling. Figure 5F shows that both rhPF4 and platelets increased sTGFBRIII release by HuT-78 cells, and that inhibition of TGFβ signaling by a TGFBRII-blocking antibody further elevated sTGFBRIII. These findings prove that PF4–TGFBRIII ligation enhanced sTGFBRIII release, and suggest that sTGFBRIII may be another important regulator of TGFβ signaling in the context with PF4–TGFBRIII interactions.

TGFBRII is the signal initiator of TGFβ1 signaling, and our above findings highlighted the importance of the interplay among PF4, TGFBII, and TGFBIII in Tn cell TGFβ signaling. We next monitored TGFBRII expression under different conditions. Platelet co-cultures increased TGFBRII surface expression of Tn cells, but the increase was diminished by blocking platelet–Tn cell contact with a transwell culture setting (Fig. 5G(a)). Similarly, incubation of HuT-78 cells with platelets elicited a dramatic elevation of TGFBRII expression, which was diminished by pre-digestion of GAGs with heparinase or/and chondroitinase (Fig. 5G(b)). These results indicated that platelet-enhanced Tn TGFBRII expression was operated via interactions with Tn cell surface GAGs in a cell-to-cell contact-dependent manner. Furthermore, rhPF4 enhanced TGFBRII expression in the presence of TGFβ1 and IL-2, and the enhancement was exerted via TGFBRIII, because the effect was abolished in ΔTGFBRIII HuT-78 cells (Fig. 5G(c)). Such an effect of PF4–TGFBRIII ligation was not a consequence of increased TGFβ signaling, as TGFBRII expression was suppressed by stimulation with rhTGFβ1 and rhIL-2 (Fig. 5G(c)).

Collectively, our results supported the notion that PF4 directly binds to GAG-rich TGFBRIII, which substantially strengthens the TGFβ signaling via increasing expression of TGFBRII. Moreover, the enhancement by PF4 requires direct cell–cell contact.

### High-concentration PF4 acts as a brake of TGFβ signaling via direct binding to TGFBRII

**Fig. 6:**
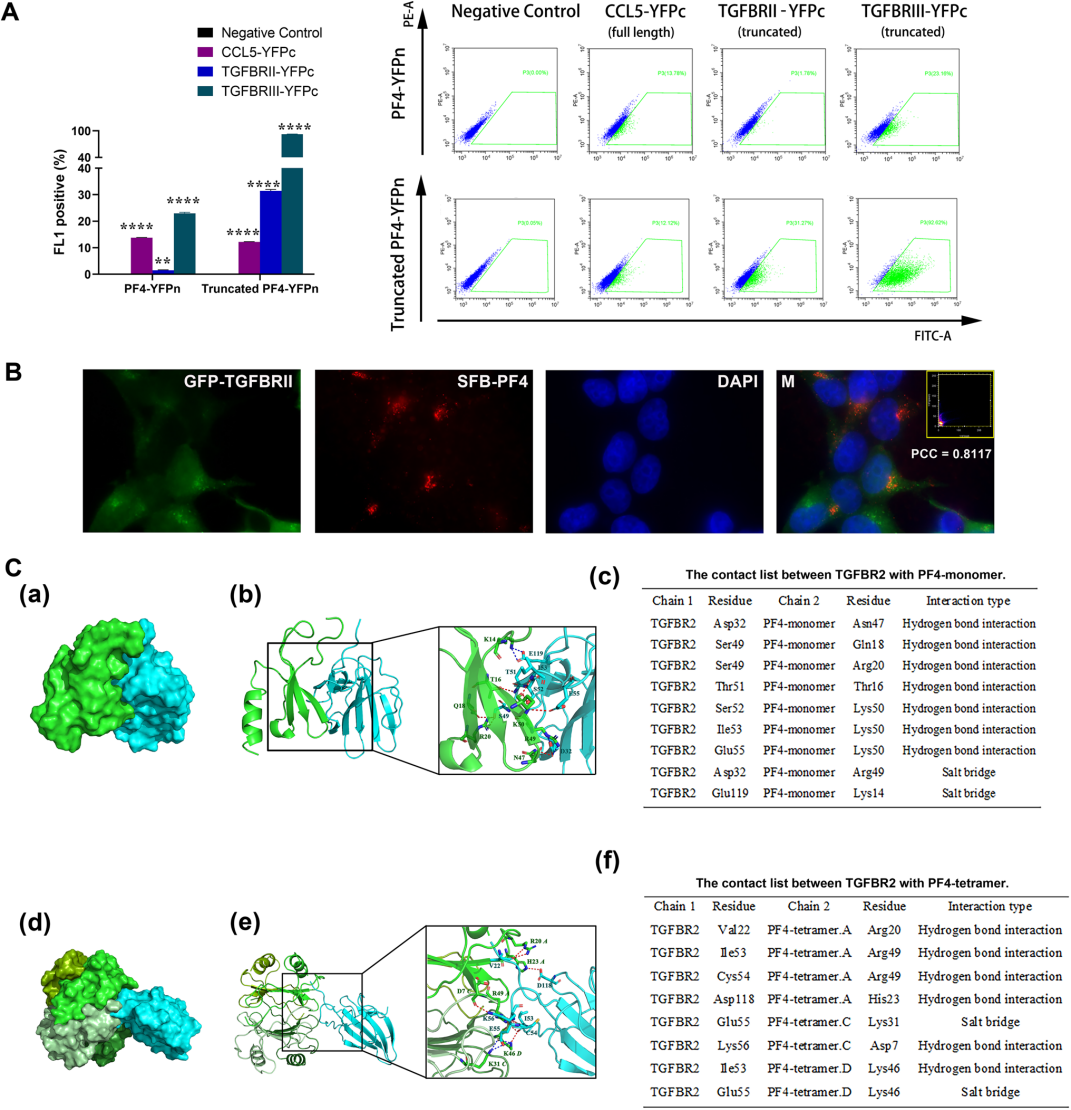
Context-dependent PF4 interaction with TGFBRII. **A** HTC-75 cells expressing full-length PF4 NYFP or truncated PF4-NYFP and simultaneously expressing CYFP-tagged CCL5/RANTES (positive control)/TGFBRII (aa 23–166)/TGFBRIII (aa 21–787)/non-fused CYFP (negative control) were phenotyped for their yellow fluorescence signals; *n* = 3; two-way ANOVA followed by Dunnett´s multiple comparisons test were performed. **B** The localizations of TGFBRII and PF4 were revealed by immunostaining. 293 T cells were transfected with plasmids encoding GFP-tagged TGFBRII and with plasmids encoding SFB-tagged PF4. Nuclei were visualized by DAPI staining. M, merged. Scatterplot of red and green pixel intensities was shown on the upper right corner of the merged picture. Pearson’s correlation coefficient (PCC) and scatterplot were calculated using ImageJ software from the compressed pictures. **C** TGFBRII-PF4-monomer and TGFBRII–PF4 tetramer docking simulations were conducted using ClusPro server. The binding mode of TGFBRII (cyan)–PF4 monomer (green) was presented in plot (a) and (b), and the contact list was in panel (c). The surface binding model of and the detailed interactions between TGFBRII (cyan)–PF4 tetramer were presented in plot (d) and (e), respectively, and the contact list was in plot (f)

The above results explained how PF4 enhanced TGFβ signaling of Tn cells at lower concentrations (0.2–5 µg/ml). The observations did not, however, explain why high concentration (25 µg/ml) of PF4 diminished the enhancements seen with lower PF4 concentrations, or why PF4 deficiency enhanced but did not suppress TGFβ1 signaling. Those phenomena indicated that there may be an inhibitory mechanism in PF4-regulated TGFβ1 signaling. To testify the hypothesis, we created a cell model with high intracellular PF4 levels by designing a truncated PF4–YFPn vector encoding the truncated PF4 with 1–31 aa signal peptide deleted [39, 40]. The truncation deleted the signal sequence for transmembrane transport, blocked PF4 packaging into α-granules, prohibited PF4 secretion, and thus allowed PF4 accumulation in cytoplasm [39–41]. Notably, transmembrane transport signal sequence is removed by proteolysis upon PF4 packaging into α-granules [41]. Hence, the truncated PF4 contained in the cytoplasm should have the same or a very similar protein structure of secreted/mature PF4, and should likely reserve a normal PF4-binding property to TGFβ receptors. Our results showed that intact PF4, i.e., with low intracellular PF4 levels, had a weak binding to TGFBRII (truncated form, aa23-166), but that truncated PF4, with an extra high intracellular concentration, had a significant increase in its ligation with truncated TGFBRII (Fig. 6A). Unexpectedly, the heteromerization of truncated PF4 to TGFBRIII (truncated form, aa 21–787) was further enhanced along with increased intracellular PF4 expression.

To further demonstrate that PF4 direct co-localizes with TGFBRII, TGFBRII-C-SFB and PF4-N-GFP were co-expressed with 293 T cells. Confocal microscopic observation showed that majority of PF4 fluorescence was co-localized with TGFBRII fluorescence (Fig. 6B).

To further elucidate PF4–TGFBRII interaction, we performed molecular docking simulations for TGFBRII–PF4 monomer and TGFBRII–PF4 tetramer. The docking (surface model/Fig. 6C(a); ribbon model/6C(b)) showed that the interactions between TGFBRII (cyan) and PF4 monomer (green) include 7 hydrogen bonds and 2 salt bridges (Fig. 6C(c)). TGFBRII–PF4 tetramer intermolecular dockings (Fig. 6C(d), (e)) engages 8 hydrogen bonds and 2 salt bridges (Fig. 6C(f)). The predicted model demonstrated good binding of TGFBRII to both PF4 monomer and tetramer. The intermolecular interactions, however, mainly involve a few hydrogen bonds that may explain the need of high PF4 concentrations for stable PF4–TGFBRII ligation. This characteristic has constituted the PF4 feature of high concentration-dependent inhibition of TGFβ signaling.

### Platelet-bound PF4 plays pivotal regulatory roles in Tn cell responses

**Fig. 7:**
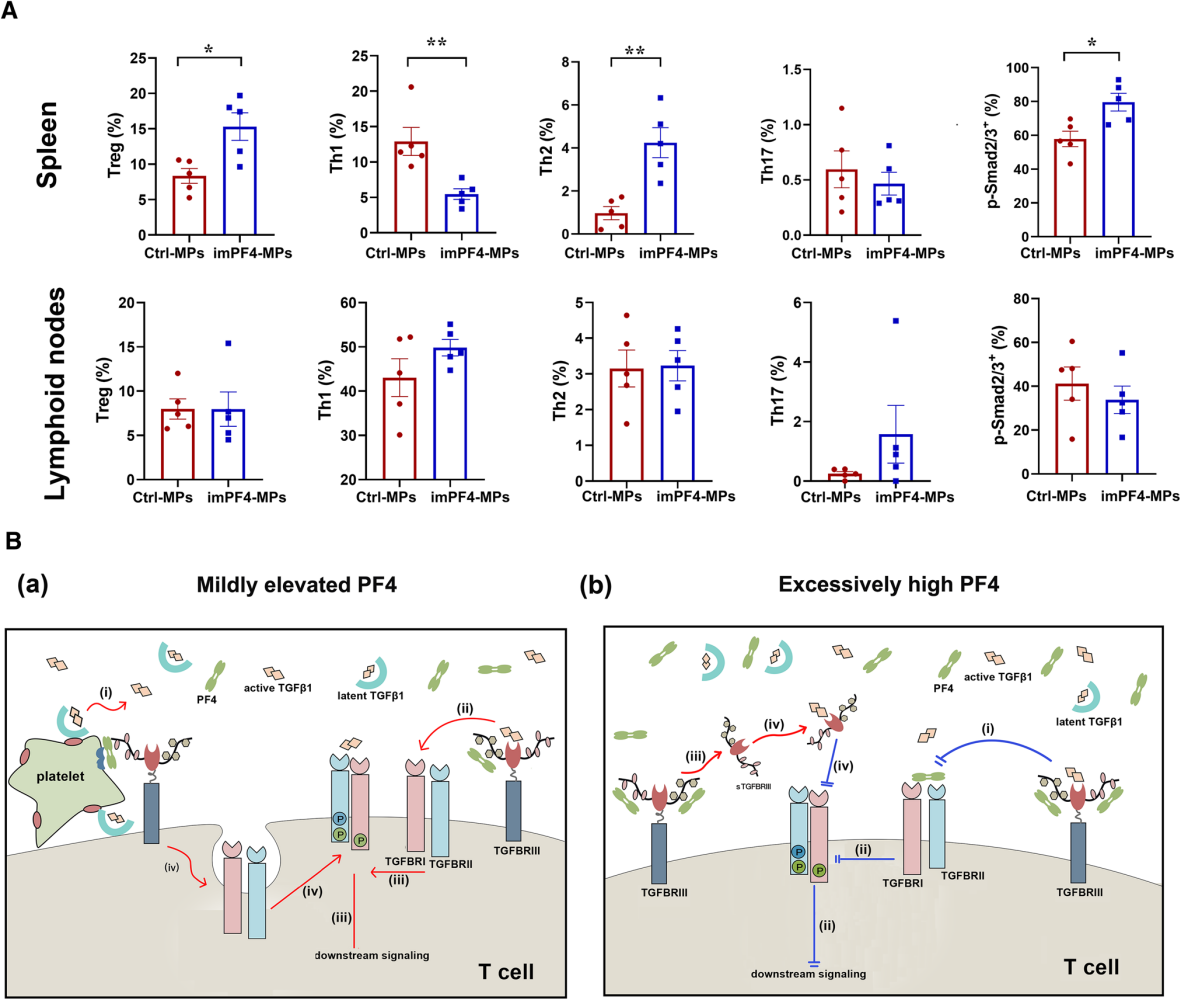
Surface-bound PF4 constitutes context-dependent platelet regulation on Tn cell responses. **A** C57BL/6 mice received OT-II T cell adoptive transfer and OVA immunization. One day after, rhPF4-immobilized aminated microspheres (imPF4-MPs) or control MPs were injected into the spleens of the mice. After a 1-week incubation, mice were sacrificed, and mononuclear cells were isolated from the spleen and the lymph nodes. CD4^+^ T effector cell phenotyping and Smad2/3 phosphorylation of OT-II T cells were assessed by flow cytometry. Data are presented as mean ± SEM, *n* = 5; Mann–Whitney test were applied between groups. **B** Schematic illustration of context-dependent platelet regulation on TGFβ signaling of naïve CD4^+^ T cells. (a) Activated platelets release and bind PF4 and latent TGFβ, and conjugate with Tn cells. Platelet-expressed molecules, e.g., GPRP/glycoprotein A repetitions predominant, facilitate the transformation of latent TGFβ1 into its active form (i), resulting in elevated levels of active TGFβ and enhanced TGFβ signaling. PF4 is capable of binding TGFBRIII via its GAG side chains. PF4–TGFBRIII ligation (ii) enhances presentation of TGFβ to TGFBRII, and (iii) increases TGFBRI/TGFBRII polymerization and downstream signaling. PF4–TGFBRIII ligation also (iv) promotes TGFBRI/TGFBRII expression of Tn cells, probably via influencing intracellular trafficking or recycling of TGFFBRI and TGFBRII to cell surface. (b) In the presence of excessive PF4, PF4 directly binds TGFBRII, which masks the binding site for TGFβ, and (ii) hampers TGFBRI/TGFBRII polymerization and downstream signaling of TGFβ. Moreover, PF4–TGFBRIII ligation (iii) increases the release of soluble TGFBRIII (sTGFBRIII). The latter (iv) competitively binds active TGFβ in the milieu, and subsequently hampers TGFβ–TGFBRII ligation and downstream signaling

Direct platelet–Tn cell contact was critical for both platelet-regulated T effector cell responses (Fig. 2C) and TGFBRII expression of Tn cells (Fig. 5G(a)). As PF4 is released from α-granules upon platelet activation, binds on platelet surface, and can facilitate the transfer of platelet-derived mediators to platelet-conjugated cells [3], we deduced that platelet–Tn cell contact and platelet surface-bound PF4 might have major impacts on TGFβ1 signaling and Tn cell activation. To testify such a hypothesis, we immobilized PF4 on the surface of the microparticles to mimic the function of PF4-bound platelets. One day after OVA immunization, PF4-immobilized microparticles (imPF4-MPs) or control microparticles were injected into the spleens of OT-II T cell adoptive transferred PF4 knockout mice. One week after the immunization, splenic and lymphatic mononuclear cells were collected for T cell phenotyping. As shown in Fig. 7A, splenic injection of imPF4-MPs markedly increased Treg/Th2 cell responses and Smad2/3 phosphorylation, but suppressed Th1 cell responses. In contrast, CD4^+^ T effector responses of LN-resided OT-II T cells were not influenced. These pieces of in vivo evidence further support the notions that platelets regulate effector responses of Tn cells through both soluble mediators and direct cell–cell contact, and that execution of the regulation requires close co-operations among soluble mediators, namely PF4 and TGFβ, and direct platelet–Tn cell contact, including platelet-bound PF4.

## Discussion

The present study has revealed a novel context-dependent platelet regulation on CD4^+^ T effector cell responses of Tn cells through close cooperation of PF4 and TGFβ (Fig. 7B). Platelets exerted their first context-dependent on Tn cell responses referring to PF4 concentrations. Thus, PF4 at low concentrations bound to TGFBRIII, enhanced TGFβ signaling and thus CD4^+^ T effector cell responses. PF4 at high concentrations, however, directly bound to TGFBRII, inhibited TGFβ-TGFBRII ligation, and subsequently exerted opposite regulatory activities. The second context-dependent regulation by platelets referred to microenvironment-dependent distinct regulation in the spleen and lymph nodes. Platelets demonstrated more profound regulation CD4^+^ T effector cell responses of Tn cells in the platelet-present, i.e., the presence of platelet–T cell contact, spleen than in the platelet-absent lymph nodes. Moreover, platelet co-cultures and PF4 enhanced sTGFBRIII release, adding a further inhibitory mechanism of TGFβ signaling and CD4^+^ T effector cell responses of Tn cells. The findings highlight a novel and sophisticated platelet regulation on T cell immunity.

The first level of context-dependent regulation referred to PF4–TGFβ signaling duet. At low concentrations, PF4 bound to TGFBRIII, promoted TGFβ signaling, and subsequently enhanced TGFβ-dependent CD4^+^ T effector cell responses, e.g., Treg/Th17 cell responses. At high concentrations, however, PF4 directly bound to TGFBRII, masked the binding site of TGFβ, thus diminished TGFβ signaling and Treg/Th17 cell responses. Such a context-dependent regulation by PF4 was achieved by the differential affinities of PF4 to the TGFβ receptors TGFBRII and TGFBRIII. PF4 bound to TGFBRIII with a high affinity, which supported significant PF4 binding already at low PF4 concentrations, and with an affinity stronger than that to CCL5 (Fig. 5A), a well-known chemokine for PF4 hetero-dimerization [31, 42]. PF4 binding to TGFBRII, on the contrary, was limited at low PF4 concentrations, and only became significant in the presence of high concentrations of PF4 (Fig. 6A), which reduced TGFβ signaling and Treg cell responses. Such delicate interactions between PF4 and TGFβ receptors ensure that TGFβ signaling is dynamically controlled depending on the local concentrations of PF4. Therefore, at the sites and probably at early stages of inflammation/immune response, massive PF4 release by activated platelets, which may also be reinforced by macrophages and T cells after activation [18, 43], increases the local PF4 concentrations to a high level. The latter enables PF4 binding to TGFBRII, as evidenced by the BiFC assay, and by docking at Asp32, Ser49, Ile53, Cys54, and Glu55 of TGFBRII (Fig. 6C). Because those sites also function as TGFBRII ligating sites to TGFβ [44], PF4-TGFBRII ligation should hinder TGFβ ligation, and can thus serve as a brake of TGFβ signaling. Indeed, PF4 neutralization and PF4 deficiency enhanced TGFβ-dependent regulation of Treg/Th1/Th17 responses and signaling in the present study. The inhibitory mechanism suppresses TGFβ signaling, attenuates TGFβ-dependent Treg cell response, and may therefore promotes swift and robust inflammatory reactions to stimuli. As the inflammation proceeds, local PF4 concentrations decrease due to diffusion and consumption, and high concentration-dependent PF4-TGFBRII binding and its masking effect diminish. Subsequently, PF4–TGFBRIII binding and its potentiating effects on TGFβ signaling take the lead, and enhance Treg cell responses that result in enhanced anti-inflammatory Treg cell activation and resolution of inflammation. To the best of our knowledge, this is the first observation showing that PF4 serves as a context-/concentration-dependent regulator of inflammation/immune responses.

The second level of context-dependent regulation referred to the lymphoid microenvironment and platelet presence, i.e., direct platelet–T cell contact. It has been well established that direct platelet-leukocyte contact is essential for the optimal effects of platelet-regulated leukocyte, including CD4^+^ T cells, functions [9, 45]. Hence, blockade of direct cell-to-cell contact by transwell co-cultures reduces platelet-enhanced CD4^+^ T effector cell responses [12]. Intercellular contact via multiple ligand-receptor systems, e.g., P-selectin–P-selectin glycoprotein ligand-1/PSGL-1 [46], CD40–CD40 ligand/CD40L [47], glycoprotein Ib/GPIb–CD11b/Mac-1 [48], and GPIIb/IIIa–fibrinogen–Mac-1 ligation [10, 12], are involved in platelet-regulated T cell functions. The present work has brought about further evidence highlighting the importance of platelet–Tn cell contact. Therefore, platelet depletion influenced T cell responses much more profoundly in the spleen, where both platelet–Tn contact and platelet-released mediators are in action, than in the lymph nodes, in which platelets, thus platelet–Tn cell contact, are absent. Consistently, platelet–Tn cell transwell co-cultures achieved only partial platelet enhancements of direct co-cultures. Moreover, platelet-specific TGFβ deficiency had more marked effects on CD4^+^ T effector cell responses in the lymph nodes than in the spleen, in which splenic platelet–T cell contact may partially compensate the loss of regulatory effects by TGFβ. These data indicated that both platelet-released soluble mediators and cell–cell contact were needed for a full action of T cell regulatory effects of platelets. Notably, platelets bind PF4 on the surface [49], and may present surface-bound PF4 to immune cells via cell-to-cell conjugation [3]. Hence, using the mouse model of OT-II T cell adoptive transfer in PF4-deficient mice, it was shown that spleen injection of control microparticles had no influence on Th cell responses in the spleen and the lymph nodes, but that spleen injection of PF4-immobilized microparticles evoked much more profound enhancements on Treg cell response and p-Smad2/3 signaling in splenic T cells than those in the lymph nodes. Our findings were supported by the observations showing that platelet–T cell conjugation attenuated Th1 and Th17 responses [50], and that blockade of platelet–T cell interaction by P-selectin inhibition reversed Th1/Th17 inhibitory effects of platelets [50, 51].

In conclusion, platelets exert context-dependent regulation on effector responses of Tn cells via PF4–TGFβ duet. With mildly elevated PF4 levels, PF4, acting as an “accelerator”, binds to TGFBRIII, facilitates TGFβ-signaling, and thus enhances effector responses of Tn cells. With extra high PF4 concentrations, PF4, serving as a “brake”, directly binds to TGFBRII, hinders TGFβ ligation and signaling, and subsequently attenuates effector cell responses (Fig. 7B). Our findings may shape a novel strategy for platelet-targeted PF4–TGFβ duet intervention of CD4^+^ T cell immunity and inflammation in the clinical settings of CD4^+^ T cell disorders, such as atherosclerosis.

## Supplementary Information

Below is the link to the electronic supplementary material.

Supplementary file1 (DOCX 4686 kb)

## Data Availability

All data generated or analyzed during the present study are included in this published article and its supplementary information files.

## Abbreviations

CBA: Cytometric bead array
DMEM: Dulbecco's Modified Eagle Media
HT-BiFC: High-throughput bimolecular fluorescence complementation technology
IFNγ: Interferon γ
IL: Interleukin
imPF4: Immobilization of recombinant mouse PF4
imPF4-MPs: PF4-immobilized microparticles
ORF: Open reading frame
PBMCs: Peripheral blood mononuclear cells
PF4: Platelet factor 4
PGI_2_: Prostaglandin I_2_
PPI: Protein–protein interaction
PRP: Platelet-rich plasma
TGFβ: Transforming growth factor β
TGFBRI: Type I TGFβ receptor
TGFBRII: Type II TGFβ receptor
TGFBRIII: Type III TGFβ receptor
Th1 cells: Type 1 T helper cells
Th2 cells: Type 2 T helper cells
Th17 cells: Type 17 T helper cells
Tn cells: Naïve CD4^+^ T cells
TNF: Tumor necrosis factor
Treg: Cells regulatory T cells
