# Supplementary material for: Platelets fine-tune effector responses of naïve CD4+ T cells via platelet factor 4-regulated transforming growth factor β signaling
Source: Cell Mol Life Sci. 2022 Apr 18;79(5):247. doi: 10.1007/s00018-022-04279-1 (PMC9016031; doi:10.1007/s00018-022-04279-1)
Supplement: Supplementary file 1 — Supplementary file1 (DOCX 4686 kb) [file 18_2022_4279_MOESM1_ESM.docx]

Supplementary Materials:

**Platelets finetune transforming growth factor β signaling and effector responses of naïve CD4^+^ T cells via platelet factor 4-regulated TGFβ receptor activities**

Yanan Min,^1,2,3^ Long Hao,^4^ Xinguang Liu,^5^ Shuai Tan,^1^ Hui Song,^6^ Hao Ni,^1^ Zi Sheng,^5^ Natalie Jooss,^1^ Xuena Liu,^7^ Rickard E. Malmström,^8,9^ Yang Sun,^10^ Jianguo Liu,^11^ Hua Tang,^11^ Hao Zhang,^2,3^ Chunhong Ma,^10^ Jun Peng,^5^ Ming Hou,^5^ Nailin Li^1^*

^1^Karolinska Institute, Department of Medicine-Solna, Cardiovascular Medicine Unit, Stockholm, Sweden; ^2^Department of Hematology, Affiliated Hospital of Jining Medical University, Jining, China; ^3^Shandong University of Traditional Chinese Medicine, Department of Clinical Medicine, Jinan, China; ^4^Department of Oncological Surgery, Affiliated Hospital of Jining Medical University, Jining, China; ^5^Department of Hematology, Qilu Hospital of Shandong University, Jinan, China; ^6^Department of Clinical Laboratory, Affiliated Hospital of Jining Medical University, Jining China; ^7^Department of Rheumatology, Qilu Hospital of Shandong University, Jinan, China; ^8^Karolinska Institute, Department of Medicine-Solna, Clinical Epidemiology Unit, Clinical Pharmacology Group, Stockholm, Sweden; ^9^Department of Laboratory Medicine, Clinical Pharmacology, Karolinska University Hospital-Solna, Stockholm, Sweden; ^10^Shandong University Cheeloo Medical College, School of Basic Medicine, Department of Immunology and Shandong University-Karolinska Institutet Collaborative Laboratory, Jinan, China; ^1^^1^Shandong First Medical University & Shandong Academy of Medical Science, Institute of Immunology, Taian, China

**Contents:**

**1. Supplementary Tables I-II**

**2. Supplementary Figures 1-6**

**Supplementary table I. Antibody and reagent list**

| Name | Manufacturer | Clone or Catalog Number | Application |
| --- | --- | --- | --- |
| anti-human CD3ε monoclonal antibody | R&D systems | clone UCHT1 | T cell stimulation |
| anti-human CD28 monoclonal antibody | R&D systems | clone 37407 | T cell stimulation |
| recombinant human CXCL4/PF4 | R&D systems | cat#795-P4-025 | T cell stimulation |
| recombinant human TGFβ1 (human cell-expressed) protein | R&D systems | Cat#7754-BH-100 | T cell stimulation |
| recombinant human IL-2 | R&D systems | Cat#202-IL-500 | T cell stimulation |
| polyclonal rabbit anti-human TGF pan specific antibody | R&D systems | Cat#AB-100-NA | neutralization |
| human TGFβ1 Duoset ELISA kit | R&D systems | Cat#DY240 | ELISA |
| DuoSet ancillary reagent kit 1 | R&D systems | Cat#DY007 | ELISA |
| human CXCL4/PF4 DuoSet ELISA | R&D systems | Cat#DY795 | ELISA |
| recombinant mouse CXCL4/PF4 | R&D systems | Cat#595-P4-025 | *in vivo* experiment |
| sample activation kit 1 | R&D systems | Cat#DY010 | TGF-β activation |
| human TGF-beta RIII DuoSet ELISA | R&D systems | Cat#DY242 | ELISA |
| Mouse TGF-beta 1 DuoSet ELISA | R&D systems | DY1679 | ELISA |
| goat anti-human TGF-beta RII polyclonal antibody | R&D systems | Cat#AF-241-NA | neutralization |
| Rabbit anti-human PF4 polyclonal antibody | PepreTech | Cat#500-p05 | neutralization |
| EasySep™ mouse CD4^+^ T cell isolation kit | STEMCELL™ Technologies | Cat#19852 | *in vivo* experiment |
| Dynabeads™ human T-activator CD3/CD28 for T cell expansion and activation | Gibco/Thermo Fisher Scientific | Cat# 11131D | Hut  -78 cell activation |
| Polybead® amino 0.50 micron micro-spheres | Polyscience Inc. | Cat# 07763-5 | *in vivo* experiment |
| glutaraldehyde kit for amino beads and blue dye beads | Polyscience Inc. | Cat#19540-1 | *in vivo* experiment |
| BD™ cytometric beads array (CBA) human/mouse Th1/Th2/Th17 kits | BD Biosciences | Cat# 560484 | cytokine detection |
| Heparinaes III | Sigma-Aldrich | Cat#H8891-5UN | *in vitro* experiment |
| Chondroitinase ABC | Sigma-Aldrich | Cat#C2905-2UN | *in vitro* experiment |
| albumin | Sigma-Aldrich | Cat#A5503-1G | *in vivo* experiment |
| Naïve CD4^+^ T cell isolation kit II | Miltenyi Biotec | Cat#130-094-131 | *in vitro* experiment |
| Inject alum adjuvant | Thermo Fisher Scientific | Cat#77161 | *in vivo* experiment |
| Rat anti-mouse GPIbα/CD42b antibody R300 | Emfret Analytics | R300 | *in vivo* experiment |
| non-specific rat IgG | Emfret Analytics | C301 | *in vivo* experiment |
| Tag-it Violet™ proliferation and cell tracking dye | Biolegend® | Cat#425101 | flow cytometric analyse |
| Zombie Aqua™ fixable viability kit | Biolegend® | Cat#423102 | flow cytometric analyse |
| rat anti-human CD4-PerCP/Cy5.5 | Biolegend® | Clone A161A1 | flow cytometric analyse |
| mouse anti-human CD41-APC/Cy7 | Biolegend® | Clone HIP8 | flow cytometric analyse |
| mouse anti-human IFNγ-FITC | Biolegend® | Clone 4S.B3 | flow cytometric analyse |
| mouse anti-human IL-17A-PE | Biolegend® | Clone BL168 | flow cytometric analyse |
| mouse anti-human IL-4-APC | Biolegend® | Clone 8D4-8 | flow cytometric analyse |
| mouse anti-human CD25-PE/Cy7 | Biolegend® | Clone BC96 | flow cytometric analyse |
| mouse anti-human/rat/mouse FoxP3-AF488 | Biolegend® | Clone 150D | flow cytometric analyse |
| precision count beads | Biolegend® | Cat# 424902 | flow cytometric analyse |
| mouse anti-mouse CD45.1-FITC | Biolegend® | Clone A20 | flow cytometric analyse |
| rat anti-mouse CD8a-PE/Cy7 | Biolegend® | Clone 53-6.7 | flow cytometric analyse |
| rat anti-mouse CD4-Brilliant Violet 605™ | Biolegend® | Clone L3T4 | flow cytometric analyse |
| rat anti-mouse IL-17A-PE | Biolegend® | Clone TC11-18H10.1 | flow cytometric analyse |
| rat anti-mouse IFN-γ-PE/Cy7 | Biolegend® | Clone XMG1.2 | flow cytometric analyse |
| rat anti-mouse IL-4-APC | Biolegend® | Clone 11B11 | flow cytometric analyse |
| Mouse anti-Smad2(pS465/pS467)/Smad3(pS423/pS425)-PE | BD Biosciences | Clone O72-670 | flow cytometric analyse |
| Transcription factor phosphor buffer set | BD Biosciences | Cat# 563239 | flow cytometric analyse |
| FoxP3/Transcription factor staining buffer set | eBiosciences | Cat# 00-5523-00 | flow cytometric analyse |
| intracellular fixation & permeabilization buffer set | eBiosciences | Cat#88-8824-00 | flow cytometric analyse |
| Human CXCL4/PF4 PE-conjugated Antibody | R&D systems | Clone # 170138 | flow cytometric analyse |
| Mouse anti-human TGFBRII APC | R&D systems | Clone#25508 | flow cytometric analyse |
| Polyclonal goat anti-mouse TGFBRII-PE | R&D systems | Cat#FAB532P-025UG | flow cytometric analyse |

**Supplementary Table II. List of PF4-interacting proteins identified by HT-BiFC assay.**


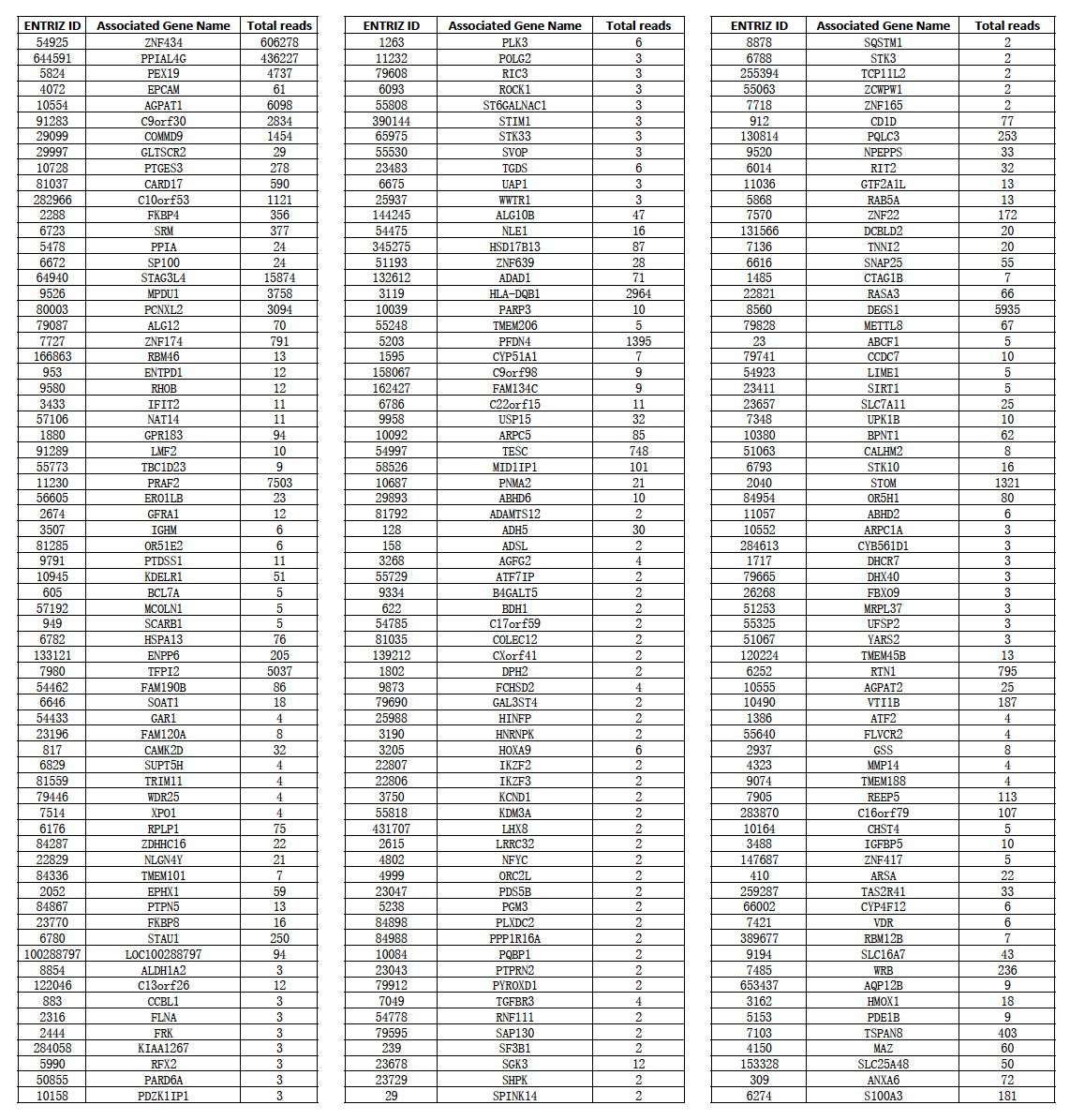


**Supplementary Figure 1**


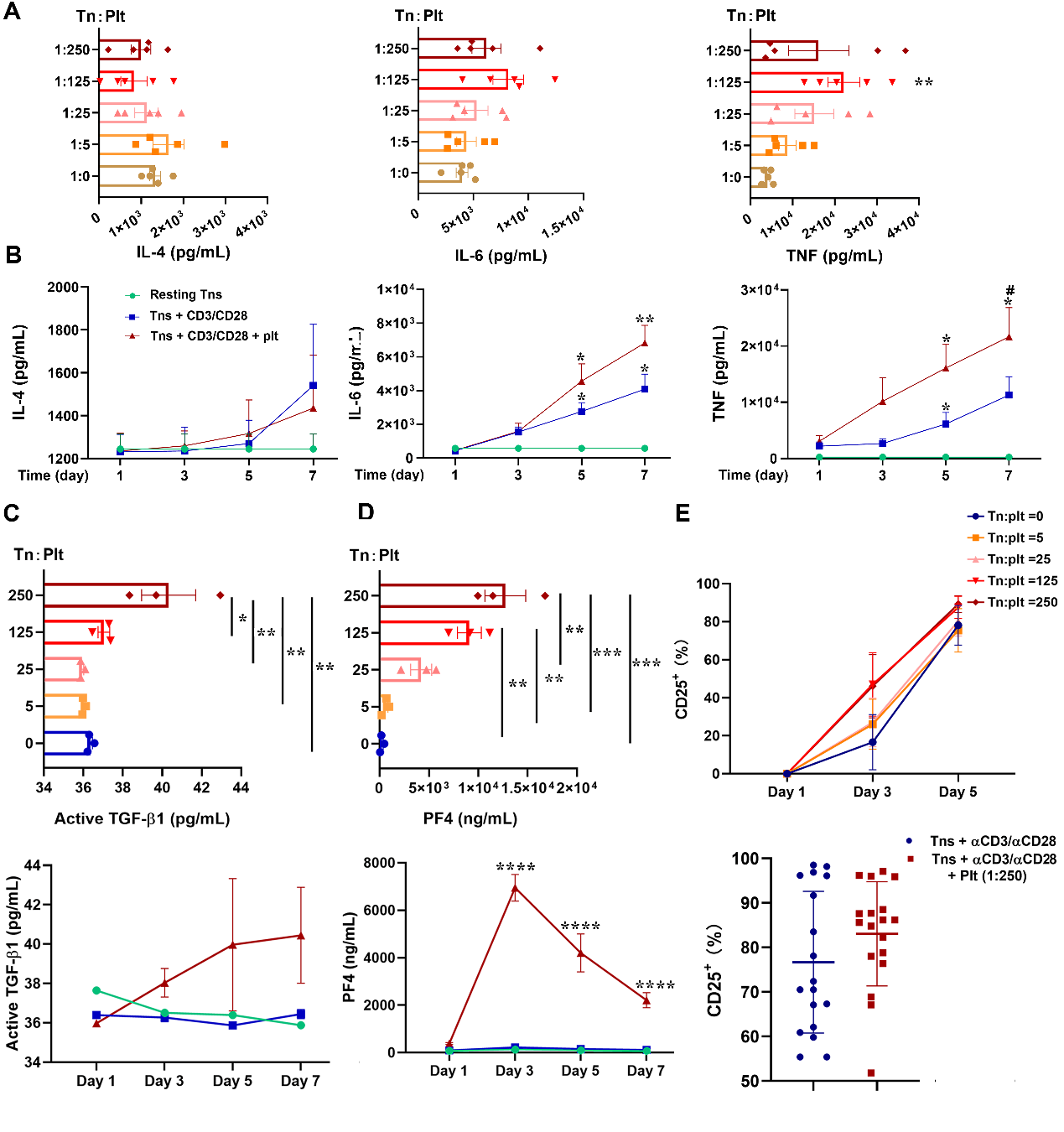


**Supplementary Figure 1. Platelet co-cultures elevate TGFβ/PF4 levels and enhance effector cell responses of naïve CD4^+^ T (Tn)** **cells.** Tn cells were cultured without or with αCD3/αCD28 stimulation and in the absence or presence of platelets (plts) at the Tn:plt ratios ranging from 1:0 to 1:250 for up to 7 days. Panel A: Tn cells were co-cultured with platelets at the Tn:plt ratios ranging from 1:0 to 1:250 for 5 days. Flow cytometric bead array (CBA) measurements of T effector cell cytokine levels in supernatants were plotted. Mean±SEM, n=5; Differences of co-culture conditions were assessed using one-way ANOVA followed by Dunnett’s multiple comparison, * *P*<0.05, ** *P*<0.01, as compared to Tn cells cultured alone. Panel B: Tn cells were cultured without (
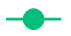
) or with αCD3/αCD28-polyclonal stimulation (
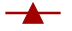
) and in the absence (
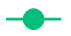

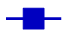
) or presence (
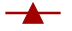
) of platelets at the Tn:plt ratio of 1:250 during 7 days. CD4^+^ T cell cytokine levels in the supernatants were measured by CBA on indicated time points. Mean±SEM, n=5. Comparisons were made by two-way ANOVA followed by Tukey’s multiple comparisons test. **P*<0.05, ***P*<0.01, as compared to unstimulated Tn cells (
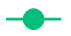
); ***#****P*<0.05, as compared to αCD3/αCD28-stimulated Tn cells cultured alone (
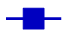
). Panels C and D: Active TGFβ and PF4 levels in the supernatant of Tn cell-platelet co-cultures were measured by corresponding ELISA kits on day 5 (upper panels) and during 7-day co-culture at Tn:plt ratio of 1:250 (lower panels); n=3. Panel E: CD4^+^ T cell CD25 expression was assessed by flow cytometry during 5 day co-cultures with different Tn:plt ratios (upper panel) and on day 5 with co-culture ratio at 1:250 (lower panel); n=18. Mean±SEM, differences of co-culture conditions were assessed using one-way ANOVA followed by Dunnett’s multiple comparison, * *P*<0.05, ** *P*<0.01, *** *P*<0.001, **** *P*<0.0001 as compared to Tn cells cultured alone or as indicated.

**Supplementary Figure 2**

**
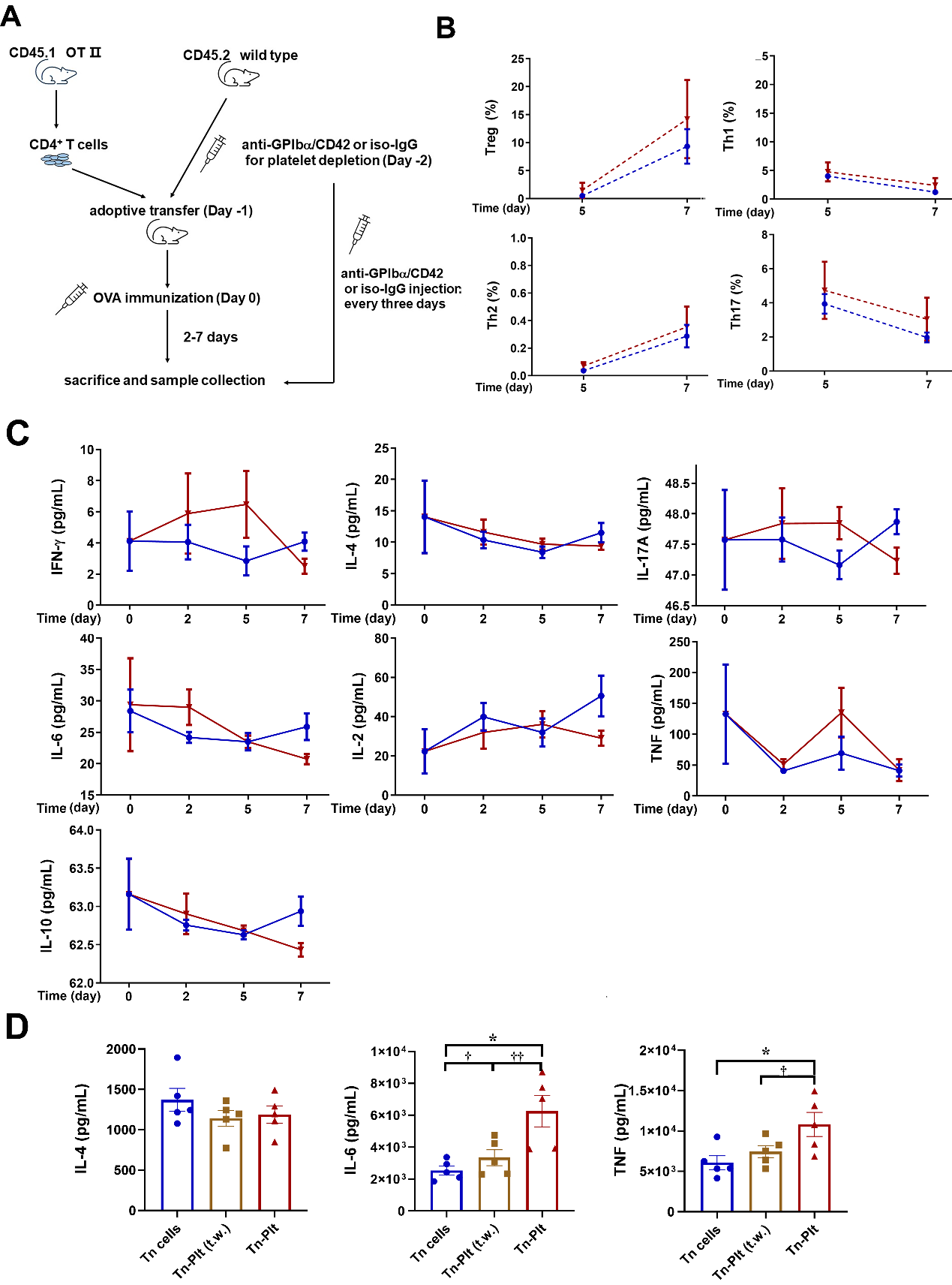
**

**Supplementary Figure 2. Platelet-regulated CD4^+^ Tn cell responses are cell-to-cell contact-dependent.** Panel A depicts the experimental design of OTII T cell adoptive transfer and platelet depletion. CD4^+^ T cells were isolated from lymphoid organs of CD45.1 OT-II mice and transferred into CD45.2 mice on day -1, followed by intraperitoneal injection of the rat anti-mouse GPIbα/CD42b antibody R300 (4 µg/g i.p. on day -2, followed by 2 µg/g i.p. on day 0, 3 and 5; blue circles and lines,
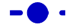
) or the non-specific rat IgG antibody C301 (red triangles and lines,
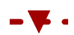
). Panel B: CD4^+^ T cells were isolated from lymphoid organs of CD45.1 OT II mice and transferred into CD45.2 mice on day -1, followed by intraperitoneal injection of the rat anti-mouse GPIbα/CD42b antibody R300 (4 µg/g i.p. on day -2, followed by 2 µg/g i.p. on day 0, 3 and 5; blue circles and lines,
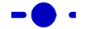
) or the non-specific rat IgG antibody C301 (red triangles and lines,
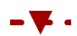
). Platelet-depleted and control mice were sacrificed on day 5 and day 7. CD4^+^ T helper cell phenotyping of mononuclear cells from the lymphoid nodes were gated on CD45.1^+^CD4^+^ T cells after eliminating cell debris and dead cells. CD4^+^ T cell phenotyping was performed for Treg/FoxP3^+^, Th1/IFN-γ^+^, Th2/IL-4^+^, and Th17/IL-17A^+^. Panel C: Plasma levels of CD4^+^ T cell cytokines were detected by a CBA assay. Panel D: CD4^+^ Tn cells were stimulated with αCD3/αCD28 antibodies, and cultured in the absence (blue circles and bars) or presence of platelets (Tn:plt = 1:250) without (red triangles and bars) or with transwell inserts (brown squares and bars; transwell membrane aperture 0.4 μm) for 5 days, and CD4^+^ T cell cytokines IL-4, IL-6, and TNF in the supernatants were assessed by a CBA assay. Data were expressed as mean±SEM; For data analyses in panel B and C, two-way ANOVA followed by Sidak’s multiple comparison test were applied. **P*<0.05, ***P*<0.01, ****P*<0.001, *****P*<0.0001. For data analyses in panel D, RM one-way ANOVA followed by Holm-Sidak’s multiple comparisons test were applied. **P*<0.05, ***P*<0.01, ****P*<0.001. Paired t-tests were also conducted between groups, †*P*<0.05, ††*P*<0.01.

**Supplementary Figure 3**

**
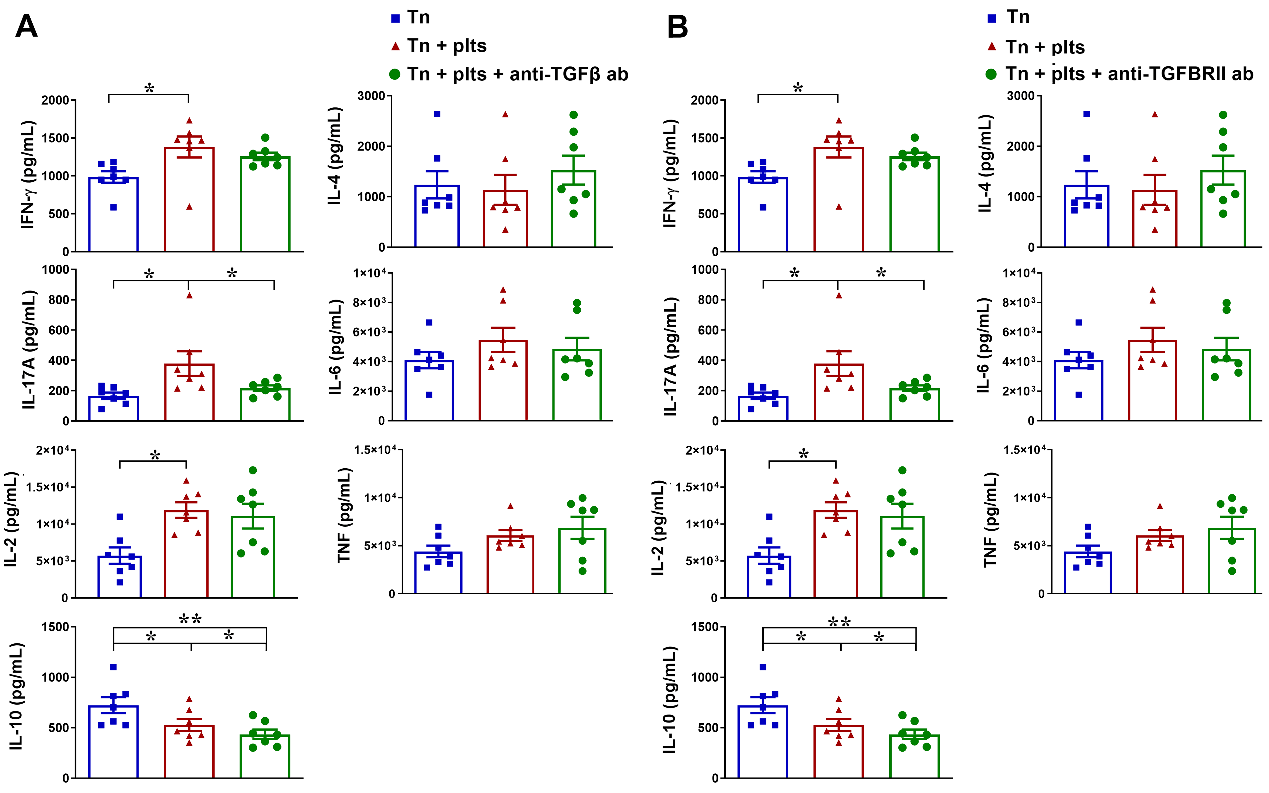
**

**Supplementary Figure 3. Concentrations of representative cytokines in the supernatants of platelet-Tn cell cocultures with/without TGFβ1 or TGFBRII blockade.** Naïve CD4^+^ T cells were stimulated with αCD3/αCD28 antibodies in the absence (blue squares
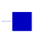
) or presence (red triangles and green circles
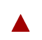

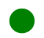
) of platelets (Tn:plt=1:250), and cultured for 5 days with (
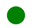
) or without (
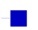

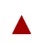
) a TGFβ neutralizing antibody (20 µg/ml; n=7; panel A) or TGFBRII blocking antibody (15 µg/ml; n=5; panel B). Representative effector T cell cytokines were assayed by CBA. Data are presented as mean±SEM. Comparisons among the treatments were performed using RM ANOVA followed by Tukey’s multiple comparison test. **P*<0.05, ***P*<0.01.

**Supplementary Figure 4**

**
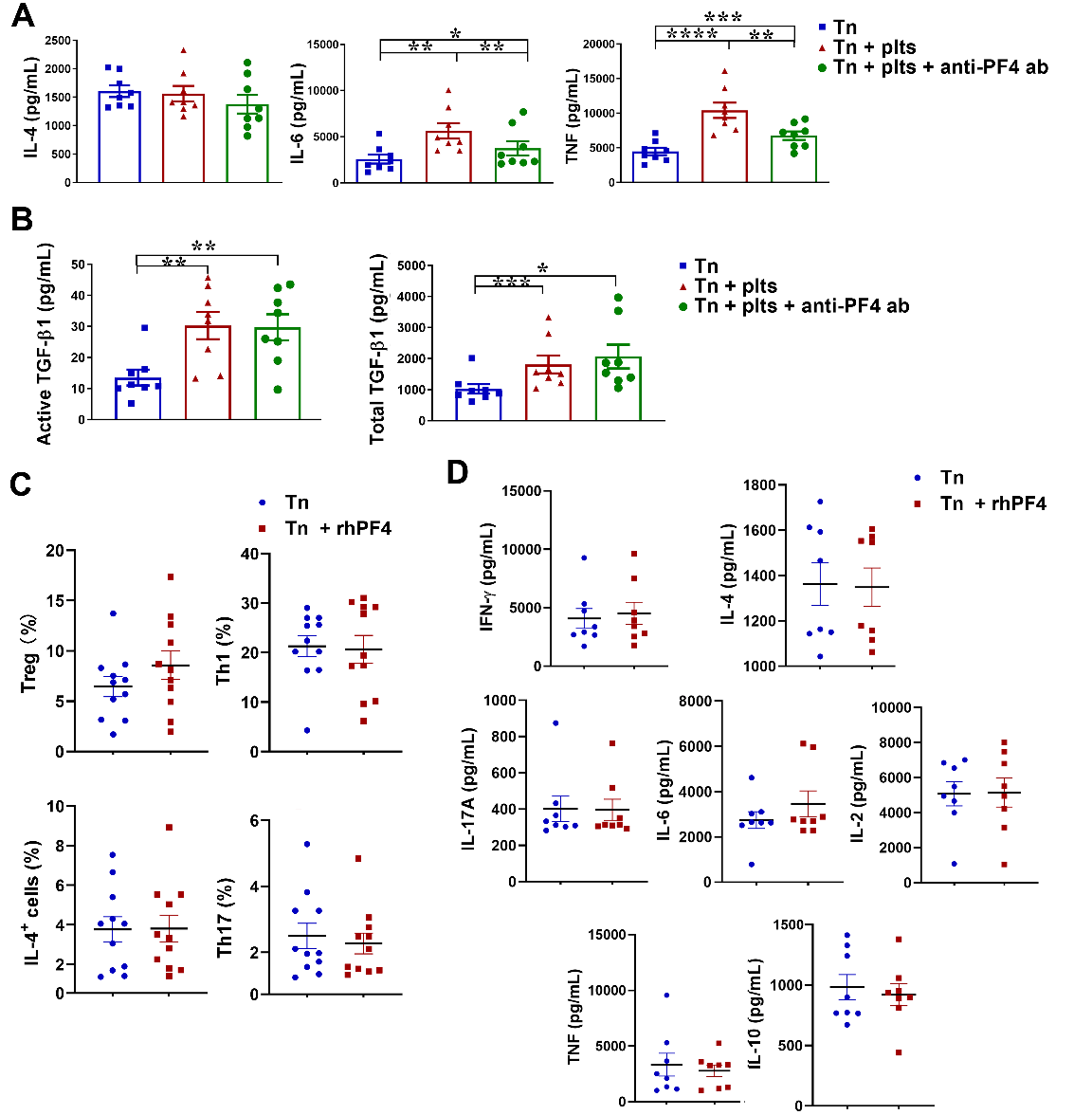
**

**Supplementary Figure 4. Impacts of PF4 on naïve CD4^+^ T cell responses**. Panels A and B: Naïve CD4^+^ T cells were stimulated with αCD3/αCD28 antibodies in the absence (blue squares
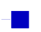
) or presence (red triangles and green circles
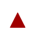

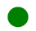
) of plts (Tn:plt=1:250), and cultured for 5 days with (
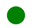
) or without (
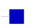

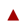
) a PF4 neutralizing antibody (25 µg/ml). CD4^+^ T cell cytokines IL-4, IL-6, and TNF (A) and the total/active TGFβ levels (B) in the supernatants were measured by CBA assay and ELISA, respectively; n=8. Panels C and D: Naïve CD4^+^ T cells were stimulated with αCD3/αCD28 antibodies in the absence (blue circles) or presence (red squares) of rhPF4 (5 µg/ml) for 5 days. Flow cytometric phenotyping of T helper cells (C) and the levels of CD4^+^ T cell cytokines (D; n=11) in the culture supernatants were assess by flow cytometry and CBA assay, respectively. Data are presented as mean±SEM. For data presented in panels A-B, the comparisons among the treatments were performed using RM ANOVA followed by Tukey’s multiple comparison test. Comparisons between the groups in panel C and D were performed using paired t-test.

**Supplementary Figure 5**


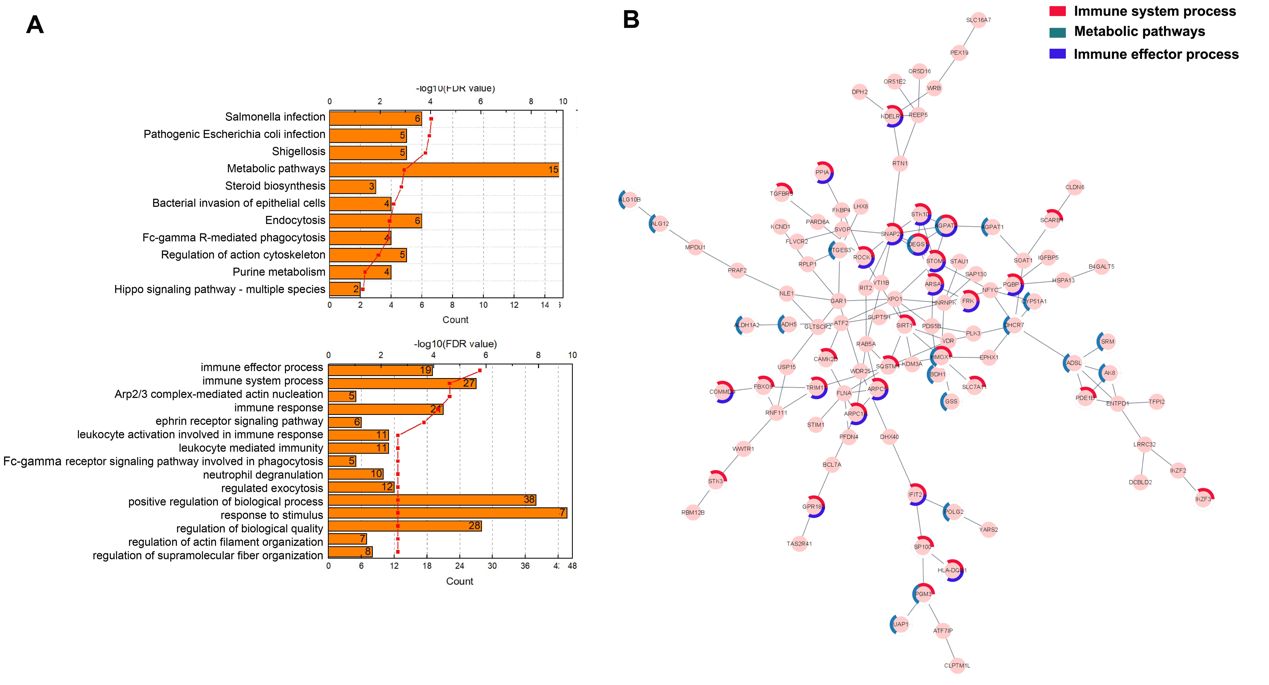


**Supplementary Figure 5. PF4 interactome analyses**. Panel A: KEGG pathway enrichment analyses were conducted using DAVID Bioinformation Resources 6.8. Top 11 enriched pathways of the PF4-interacting proteins, including their corresponding protein counts and *p*-values, are presented in the upper bar chart on the left. Top 15 PF4-interacting protein groups within the “Biological process” as assessed by GO analyses are presented in the lower panel. Panel B: Functional interactions of PF4-interacting proteins were assessed and networked with STRING. **Supplementary Figure 6**


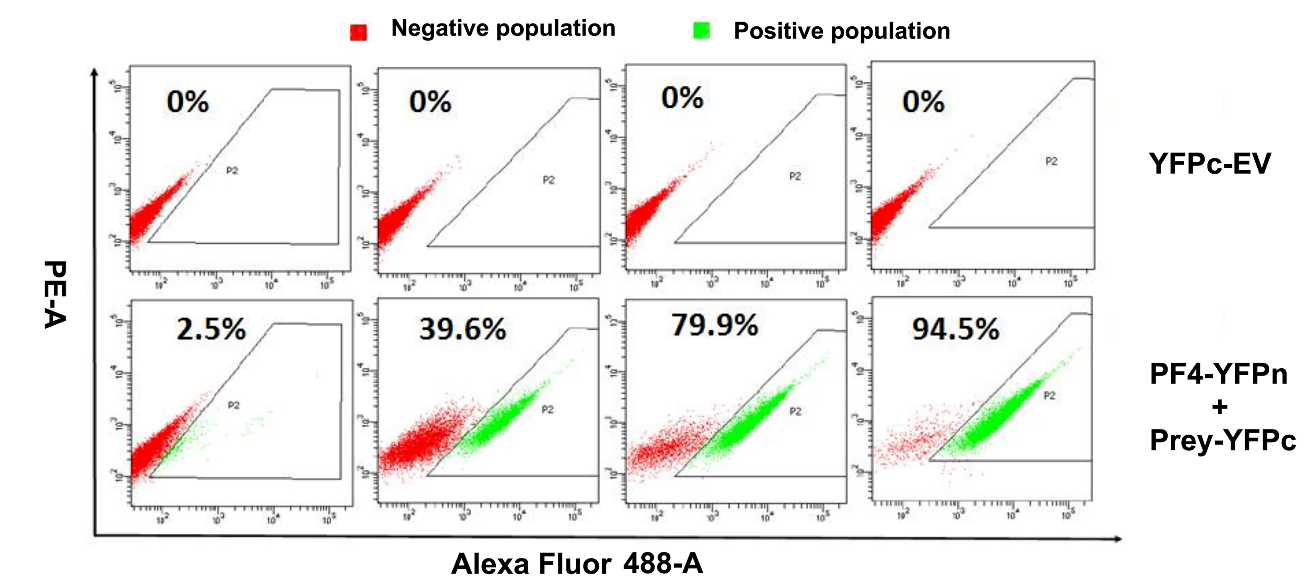


**Supplementary Figure 6. Flow cytometry diagrams of cell sorting.** Representative flow cytometric diagrams of HTC-75 cells co-expressing PF4-YFPn and Prey-YFPc or control construct EV-YFPc (EV: empty vector), showing the ultimate positive sorting rate at more than 90%. HTC-75 cells were transfected by PF4-YFPn, Prey-YFPc, or EV-YFPc. After 3-day culture, the cells were sorted for BiFC negative and positive cells. The upper panels depict the negative control in the 1-4 round of cell sorting, and the lower panels depict the corresponding PF4-YFPn and Prey-YFPc co-transfected cells. During multiple rounds of screening, the population of BiFC-positive cells of PF4-YFPn/Prey-YFPc co-transfected cells increased steadily. The positive rate of the co-transfected cells reached the level of >90% after 4 rounds of sorting/selection. The results indicated that the proteins with interaction were enriched continuously, which ensured the significant difference between desired signal and background signal. The multiple screening and sorting reduced possibility of false positive, and ensured the authenticity and credibility of the obtained data.
